# Maximum power point tracking in fuel cells an AI controller based on metaheuristic optimisation

**DOI:** 10.1038/s41598-024-83453-w

**Published:** 2024-12-30

**Authors:** P.M. Preethiraj, Belwin Edward J.

**Affiliations:** School of Electrical Engineering, Vellore institute of technology, Vellore, Tamil Nadu India

**Keywords:** Engineering, Electrical and electronic engineering

## Abstract

The increasing concern about global warming and the depletion of fossil fuel reserves has led to a growing interest in alternative energy sources, particularly fuel cells (FCs). These green energy sources convert chemical energy into electrical energy, offering advantages such as quick initiation, high power density, and efficient operation at low temperatures. However, the performance of FCs is influenced by changes in operating temperature, and optimal efficiency is achieved by operating them at their maximum power point (MPP). This study uses Proton Exchange Membrane Fuel Cells (PEMFCs) to charge electric vehicles (EVs), amplifying the voltage generated by the FC using the Interleaved Boost-Cuk (IBC) converter. The optimal tracking of the maximum power output is achieved using the Improved Mayfly optimized (IMO) Cascaded Adaptive Neuro Fuzzy Inference System (Cascaded ANFIS). The study uses MATLAB to simulate the task in various settings and analyze the relevant performances, demonstrating enhanced efficiency and power tracking outputs. The proposed converter efficiency has improved to 94% with a minimal part count of 2 switched configurations. configuration. The applied control logic, in my opinion, Cascaded ANFIS is capable of operating the BLDC with an operational efficiency of 98.92%, including better output voltage generations of 350 V.

## Introduction

Today, the availability, accessibility, and generation of sustainable electrical energy are inextricably linked to the global economy. The global ecosystem and climate change have already been profoundly damaged by conventional energy production techniques^1^. Environment-related emissions of Greenhouse Gases (GHG) will result in significant climate deterioration with an average of 6 °C global warming, according to the latest study released by the International Energy Agency^2,3^. Sustainable energy is a realistic alternative in this scenario for making the globe a safer and more resource-efficient environment to live. Because Co_2_ emissions, the most fundamental sign of the harmful greenhouse effect, are kept to a minimum with clean energy, it is a better option^4^. FCs, biogas, Energy Storage Systems (ESS), and Renewable Energy Resources (RES) are a few examples of these alternative power sources. The modular construction of FCs, which improves dependability, versatility, and adaptability to the desired amount of power generation, gives those many advantages over other alternative sources. In addition, electrolyzers is utilized to transform excess RES electricity into hydrogen fuel production, which can be preserved and utilized to power FCs when there is a shortage of power^5^. Due to the use of hydrogen fuel and ambient oxygen as inputs and a chemical process that yields electricity and water as outputs, electrolyzers, and FCs produce no pollution during operation and emit nearly no greenhouse emissions. Direct-methanol FCs^6^, Alkaline FCs^7^, Molten Carbonate FCs^8^, PEMFCs^9^, Solid Oxide FCs^10^. , and phosphoric acid FCs are only a few of the several types and approaches used to categorize FCs. PEMFCs are among them and are particularly well-liked because of their outstanding performance traits, which include quick start-up, low weight, and optimal working temperature^11^. Changes in temperature during operation, Membrane Water Content (MWC), and gaseous hydrogen oxygen pressures all affect the output in FCs. PEMFC exhibits non-linear V-I and P-I attributes with a global peak power point for variable current with constant temperature, MWC, hydrogen gas, and oxygen partial pressures^12,13^. Therefore, to increase FC efficiency, it is preferable to operate FCs at their MPP^14^. The development of a heuristic optimization method for achieving optimal MPP with reduced disturbances in a steady state is the main focus of this paper.

The Perturbation and Observation method (P&O) is typically regarded as the most popular by its simplicity and ease of use. The PEMFC’s persistent voltage and current measurements serve as the foundation for its operation, which subsequently calculates the power output. The perturbation is introduced in the opposite sense if the new value of alteration in power output is negative^15,16^. If maximum power is not obtained, this strategy continues to be searched for. From a different angle, the variable step size MPPT approaches have proven to be superior substitutes for fixed step size MPPT solutions. Incremental conductance (INC) has been used with PEMFC systems^17^. However, flaws such as delay in response, complexity towards non-linear operating, and oscillation approaches fail. To tackle the mentioned flaws, an Artificial Neural Network (ANN) MPPT is designed^18^, which can exact the most power with improved accuracy but requires high computational time, and overfitting occurs. The fuzzy logic control (FLC) MPPT approach for PEMFCs is created as a response to the aforementioned problems with existing MPPT methods. The benefits of the suggested design include quick tracking, easy deployment, and less expensive sensors^19^. Besides its benefits, imperfections while designing fuzzy rules and a lack of interpretability limitations appear. Fuzzy logic and neural networks are combined to create ANFIS-based MPPT control^20,21^, a type of artificial intelligence (AI) that combines the benefits of both techniques and results in superior performance towards tracking than other MPPT approaches. Despite generalisation to new conditions, overfitting, and training time, the system is complex. Hence, in the proposed work, a cascaded ANFIS-optimized (IMO) algorithm is proposed to further enhance the system performance of classical ANFIS-dependent MPPT techniques.

An unregulated low DC output voltage is generated by a stack of PEMFCs. To increase and control PEMFC output voltage, a boost or a step higher DC-DC converter is needed. The fuel cell makes heavy use of boost converters as front-end power conditioners. The typical boost converter serves as electronic interface for low power applications, but due to its limited capacity to handle current and thermal management difficulties, boost converters is suitable with high power solicitations^22^. To solve these issues various high voltage gain converters have been developed. In Buck-Boost converter step up (boost) or step down (buck) voltage is utilized to boost fuel cell with improved flexibility, and high efficiency, but attributes such as high stress components, control complexities leads the system to malfunction^23^. Similarly, Cuk and SEPIC converters are established for enhanced voltage regulation owing to their advantageous benefits like continuous output current, bidirectional operation, and reduced ripple^24,25^. However, lower capacity to handle power and reduced efficiency cause system frustration. Henceforth, interleaved Boost converter^26^, is designed for fuel application owing to its benefits like minimized switching loss and high voltage gain. Moreover, the fuel cell’s dependability is improved by the interleaving process, which also offers high power capabilities. Despite its advantageous, an increase in the number of components creates stress and drops system efficiency. Therefore, the proposed research is accomplished with interleaved Boost-Cuk topology, due to its benefits for interfacing a fuel cell system. The suggested converter’s output voltage is sent to vehicle’s electric motor via an inverter for propulsion. In Fuel cell electric vehicles (FCEVs), electric motor has a crucial function. The cost and size of fuel cell are significantly decreased by an adequate motor.

For the majority of their electric vehicle applications in the past, manufacturers used DC motors. DC motors, on the other hand, are less efficient and have higher maintenance costs because of the brushes and revolving parts. Permanent magnet BLDC motors are currently used mostly in FCEV applications because of their straightforward control, excellent reliability, and high robustness. Table 1 describes the existing works based of PEMFCs together with its merits and demerits. The contribution of the proposed work involves:

- Accomplishment of PEMFCs as a clean source of power generation owing to its advantages including low noise, enhanced efficiency, and improved reliability.
- Interleaved Boost-Cuk converter is established to regulate and boost fuel cell power for effective motor performance.
- Adoption of IMO-Cascaded ANFIS MPPT controller for extraction of optimal fuel cell power.
- MATLAB Simulink execution is performed to improve the efficacy of the proposed fuel cell-based EV system.
- Detailed comparative examination is executed to reveal the superiority of proposed techniques.

The rest of the paper is systematized as a description of proposed work in Section III, modeling of proposed system components at Section IV, outcomes generated by MATLAB in Section V, and conclusion in Section VI.

## Literature review

This section provides a comprehensive overview of existing literatures related to FC based EVs and MPPT system. The review focus on various optimization techniques and converter topologies utilized for enhanced performance of fuel cell.

**Table 1:** Summarizes the key studies, based on PEMFCs together with its merits and demerits.

Author/ Year of Publication/ Reference	Converter	Control Approach	Merits	Demerits
K. Kumar, et al., 2021^27^	Quadratic Boost	P&O and Neural Network MPPT	High voltage gain transfer	Decreases general proficiency of system.
Slah Farhani, et al., 2020^28^	Interleaved Boost	Space Vector Averaging	Minimized current pressure and reduced current ripple.	Increased component stress.
Balasubramanian Girirajan, et al., 2022^29^	High Gain Converter	Radian Basic Function Algorithm	Conversion ratio is improved with reduces current ripple and voltage stress.	High switching loss occurs with a slow response.
Shaik Rafi Kiran, et al., 2022^30^	Single Switch Boost	Modified Variable Step Size RBFN MPPT	Stress across power switches is low and achieves continuous power supply.	Power gain is low and requires more number of switches.
C. Shilaja, et al., 2022^31^	Boost	Improved PSO, Adaptive Cuckoo Search Optimization	Increased voltage gain ratio with good tracking speed.	Switches receive an excessive amount of current.
Salem Saidi et al., 2024^34^	Fuel cell strategy	Enhanced Salp Swarm Algorithm (ESSA)	Improved accuracy in parameter estimation, Faster convergence speed, High stability, and robustness	Complexity in implementation due to multiple enhancements It may require computational resources
Pathak, Pawan Kumar et al.,2023^35^	Fuel cell strategy	Decarbonization involves renewable energy, CCS for blue hydrogen, and electrolysis for green hydrogen, focusing on reducing fossil fuel use.	High energy storage potential, Decarbonization of hard-to-abate sectors, Enables renewable integration, Reduces CO2 emissions significantly by 2050	High initial investment, Challenges in scaling CCS and electrolyzer production, Dependence on scarce resources for electrolysis, Infrastructure development needed

## Proposed system

**Fig. 1:**
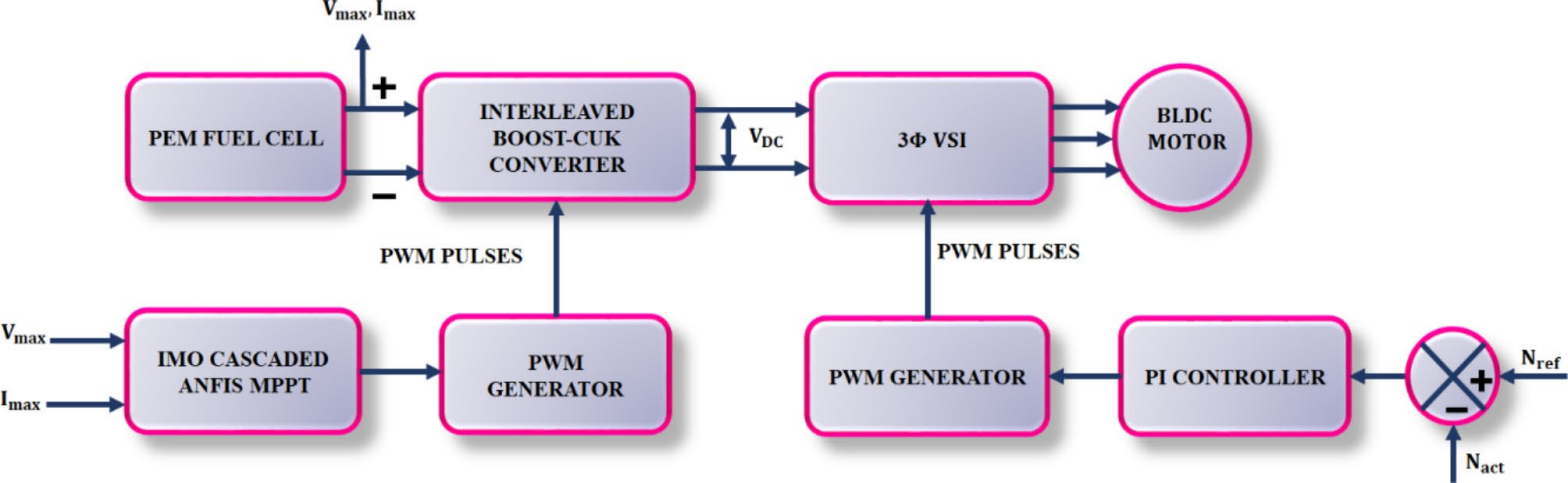
Architecture of proposed fuel based EV.

Fuel cell systems are advancing in addition to the desire to provide electricity in remote locations as well as distributed power generation, particularly during peak loads. Due to their cleanliness, portability, and adaptability for producing both heat and power, fuel cells are growing in popularity. In order to interface with loads or EV, as shown in Fig. 1, this work describes the modelling of PEM fuel cell.

In the proposed work a PEM fuel cell is utilized as a source form generating power. In general PEMFC is an electrochemical device that performs an electrochemical reaction to transform chemical energy contained in hydrogen and oxygen into electrical energy. It is made up of catalyst layers, anode and cathode electrodes, and a polymer electrolyte membrane. The energy from PEMFC is fed to converter for boosting of voltage. In this research, interleaved Boost-Cuk (IBC) converter is accomplished. The proposed converter combines the functionalities of both Boost and Cuk converter and operates in an interleaved manner, with reduces ripple at converter’s input and output side. In order to extract optimal power from PEMFC and to continuously modify the operating point for attaining the most possible output power Maximum Power Point Tracking (MPPT) is introduced. In this paper, an AI model termed cascaded ANFIS with Improved Mayfly Optimization (IMO) is employed. The usage of optimisation procedure motivated by the behaviour of mayfly insects tunes the parameters of cascaded ANFIS to extract most possible fuel cell power. Hence, stabilized DC link voltage is and is transferred to $\:3{\Phi\:}\:VSI$ for conversion of DC supply into AC together with Proportional Integral (PI) controller for effective inverter controller. Finally, the attained AC supply is transferred to BLDC motor (EV motor), in which the speed of BLDC motor is optimized using PI controller. The overall experimental validation is simulated on MATLAB platform and outcomes accomplished benefits in providing optimal system performance, enhanced power output and improved efficacy.

## Modelling of proposed system

### Pem fuel cells

**Fig. 2:**
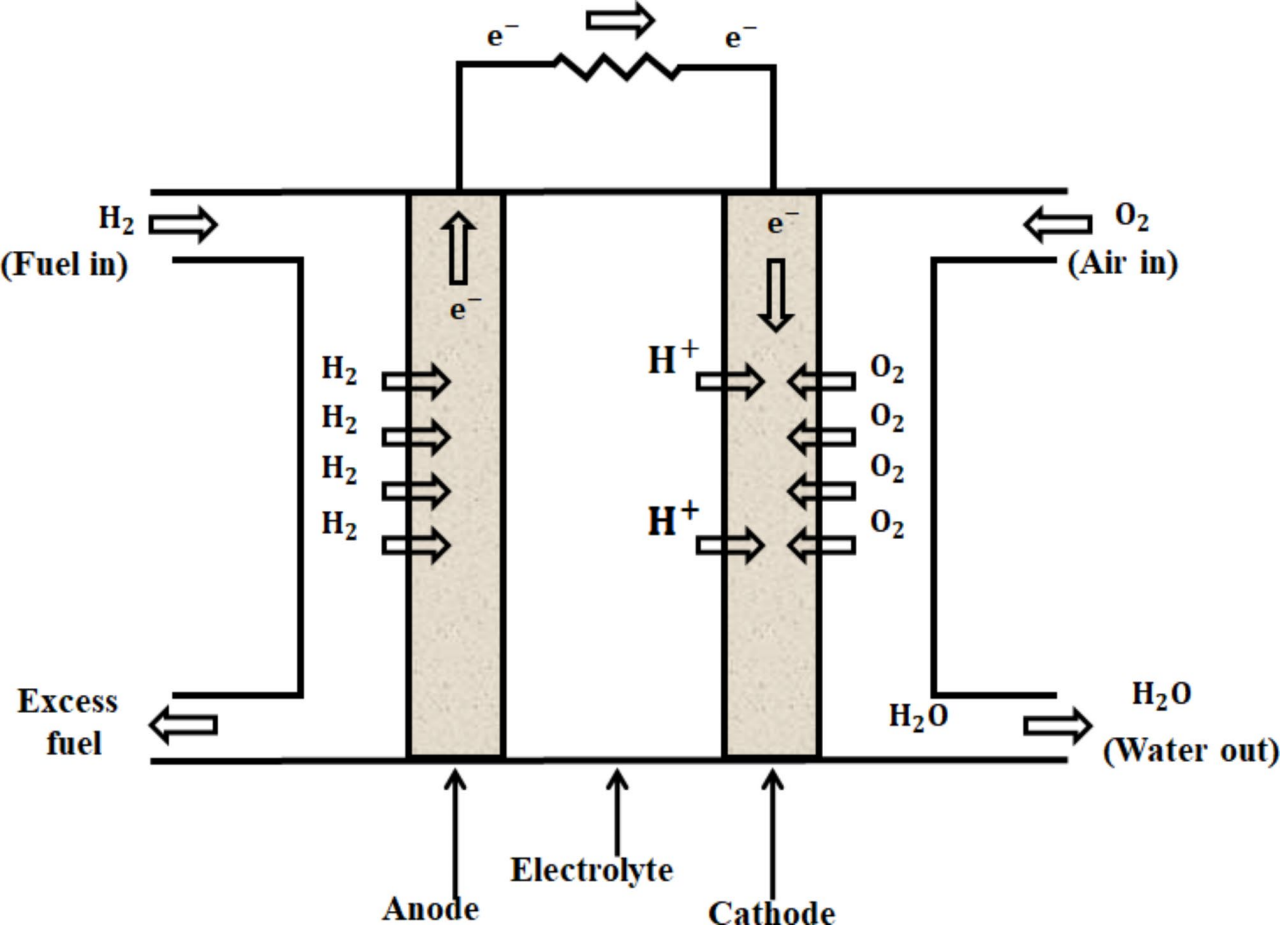
Construction of PEM fuel cell.

The PEMFC is a form of fuel cell that is becoming more and more well-liked because of its versatility, high efficiency, and power density. When compared to energy converters that use conventional fossil fuel to generate electricity, PEMFC is one of the most effective. Figure 2 demonstrates the standard PEMFC’s construction.

Hydrogen ions serve as carriers in PEMFC, moving from anode to cathode. The anode compartment is delivered with hydrogen gas. Due to chemical composition of H2, hydrogen ions $\:\left({H}^{+}\right)$ and electrons $\:\left({e}^{-}\right)$ are then separated. Then, H + travels across polymer membrane electrolyte and arrives at cathode. Since they are forbidden from passing through proton exchange membrane, electrons must flow through external circuit in order to move from anode to cathode. The cathode chamber, which has both positive $\:{H}^{+}$ and negative$\:\:{e}^{-}$, receives air at same time. Water molecules are generated when oxygen in air is combined with the ions e and$\:\:{H}^{+}$. The chemical reaction takes place during operation is as follows:

$$\:AnodeReaction:{H}_{2}\to\:2{H}^{+}+2e^-$$

$$\:Cathode\:Reaction:\:2{H}^{+}+\frac{1}{2}{O}_{2}+2{e}^{-}\to\:{H}_{2}O$$

$$\:Overall\:Reaction:{H}_{2}+\frac{1}{2}{O}_{2}\to\:{H}_{2}O$$

### Fuel stack

Since a single cell is capable of generating voltages between 0 and 1 V, which is far too little even to drive semiconductor devices adequately, multiple cells are linked in series to form a fuel cell stack. A FC generates necessary power for a small appliance, but for big power applications, it must be combined with other energy sources, including supercapacitors or batteries that can be recharged. A quick change in load current minimises generated dc voltage due to fuel cell’s internal resistance. If the system is exposed to abrupt power drops, additional storage is frequently employed in conjunction with fuel cell.

**Fig. 3:**
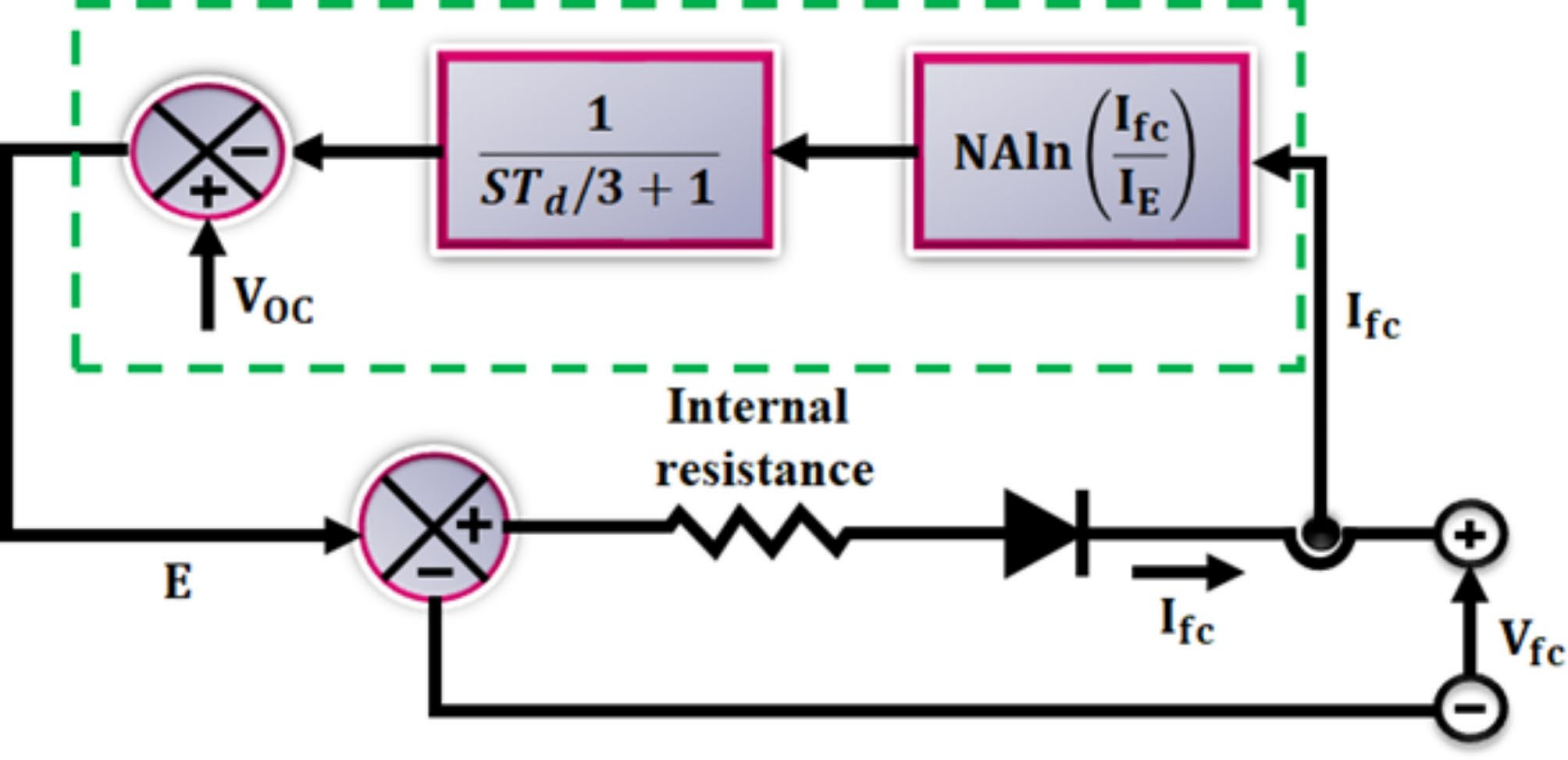
PEMFC’s equivalent circuit.

 Due to its unique qualities and a 5 to 10 times higher power density than normal batteries, FCs in convenient applications have many advantages over those of conventional batteries. From a few watts to several kilowatts, portable fuel cells produce a wide spectrum of power. Manufacturer Horizon has already released instructional remote-control toys, kits, and equipment on market, including a hydro car educational package. Electricity is produced using fuel cell-based movable power generators when a grid connection is not possible. For personal outdoor use, security, and disaster assistance, the portable generator appears to be enough. In the stationary power generation industries, fuel cell technology is important. Figure 3 depicts the equivalent circuit configuration of PEMFC. Where V_0C is used to represent open-circuit voltage, internal resistance also appears there. A diode is used in series with output to control the flow of negative current into system.

### Operation of interleaved boost-cuk converter

**Fig. 4:**
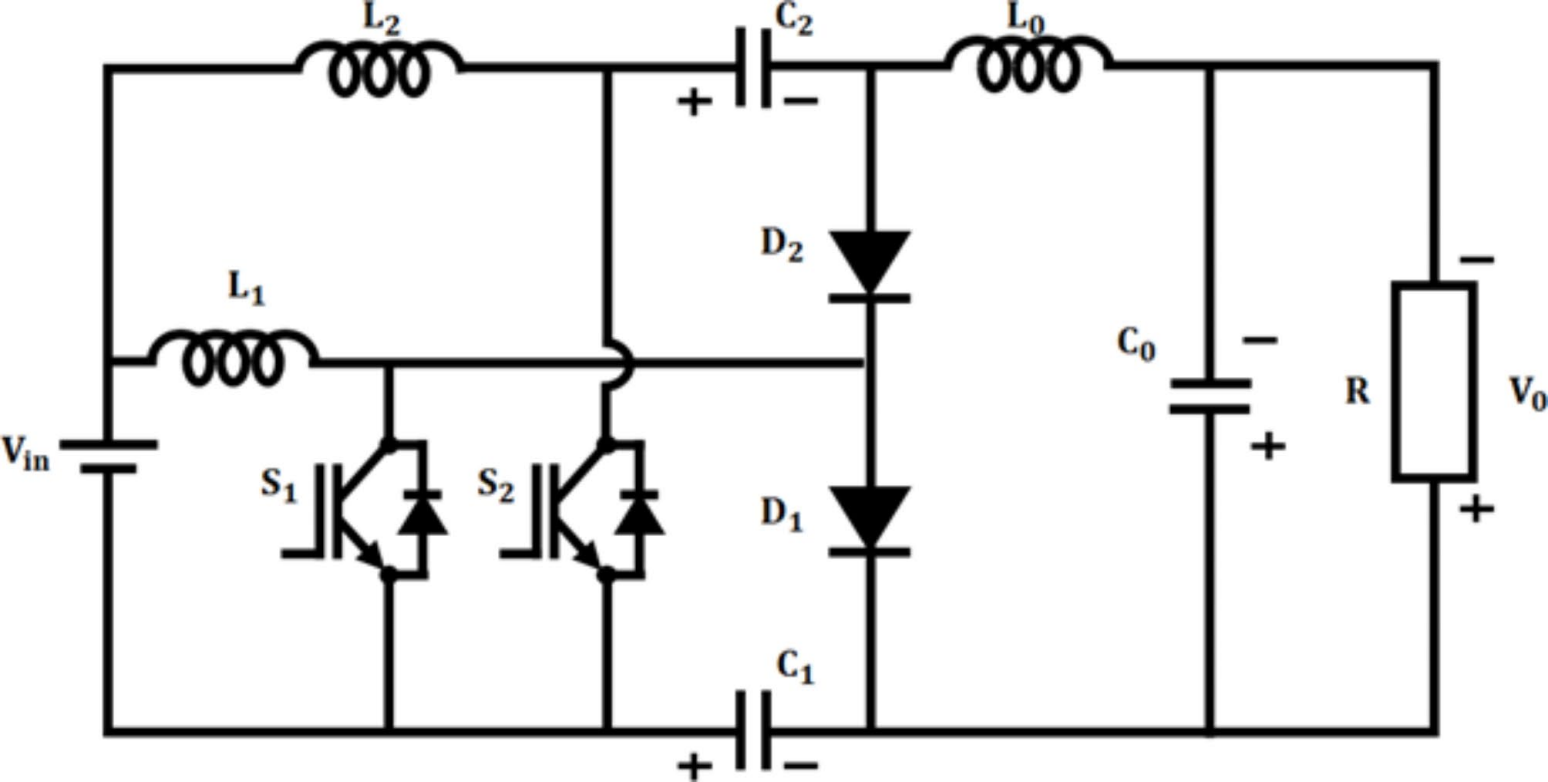
Interleaved boost-cuk converter configuration.

The voltage from fuel cell is low and is not sufficient for effective functioning of BLDC motor, which necessitates the adoption of power electronic converter. In the proposed work, interleaved Boost-Cuk converter which combines the characteristics of both boost and Cuk converter is accomplished for boosting of fuel cell voltage. Figure 4 depicts the configuration of proposed IBC converter.

### Operating modes of IBC converter

**Fig. 5:**
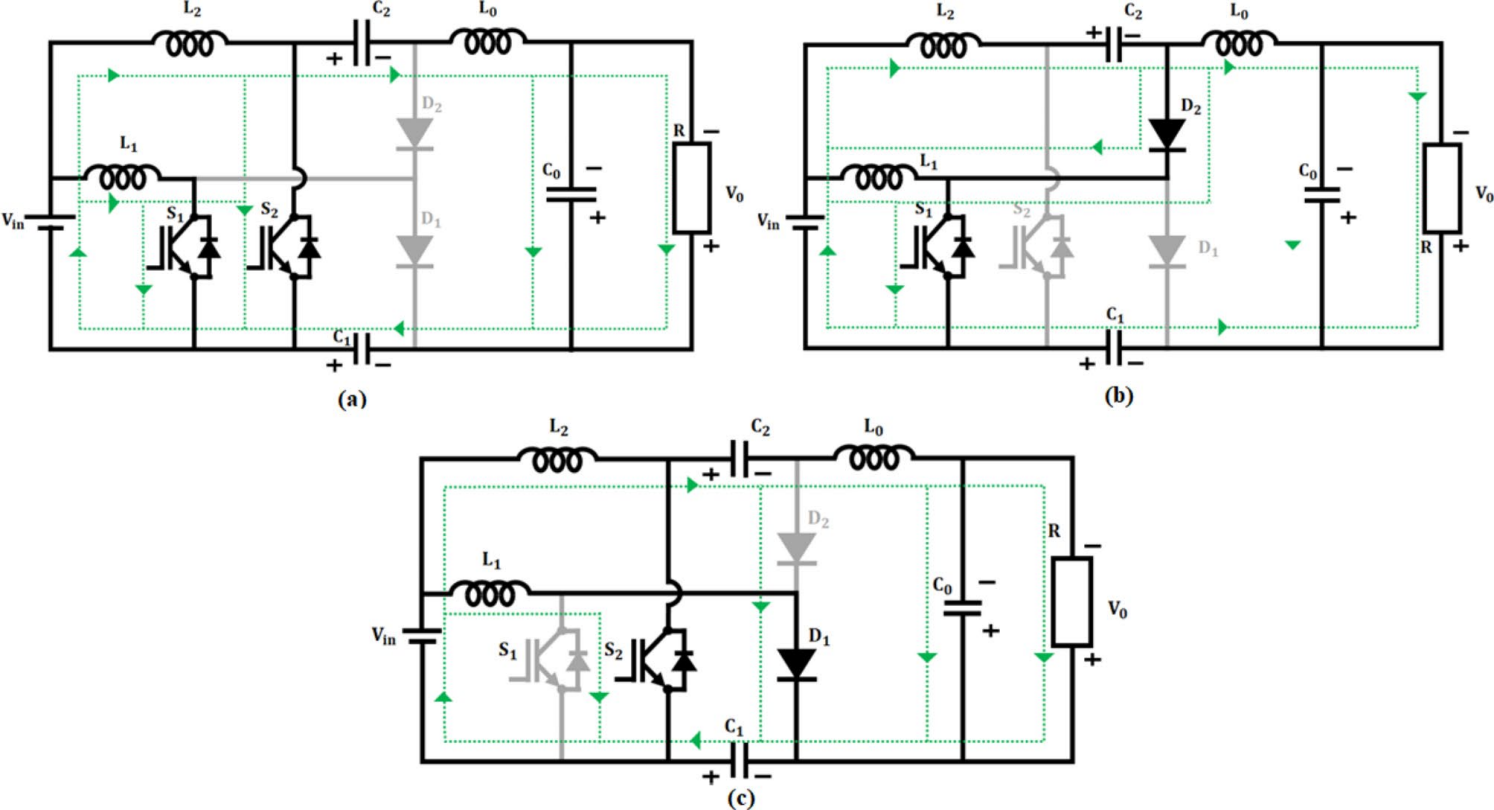
Converter operation at (**a**) Mode I, (**b**) Mode II and (**c**) Mode III.

The operating modes of interleaved boost cuk converter Fig. 5 includes 3 modes: mode I Fig. 5(a), mode II Fig. 5(b), & mode III Fig. 5(c).

$\:\varvec{M}\varvec{o}\varvec{d}\varvec{e}\:\varvec{I}\:[{\varvec{t}}_{0}-{\varvec{t}}_{1}]$: At this instance, both the switches $\:{S}_{1}$ and $\:{S}_{2}$ are in ON state, while the diodes$\:\:{D}_{1}$ and $\:{D}_{2}$ turns OFF as seen in Fig. 5(c). During this condition, the inductor $\:{L}_{1\:}$starts charging by attaining voltage from source$\:\:{V}_{in}$. Correspondingly, capacitors linked in series $\:{C}_{1}$ and $\:{C}_{2}$ discharges and charge the output inductor$\:{\:L}_{0}\:$by switch$\:\:{S}_{2}$. In accordance with this the output inductor current$\:\:{i}_{{L}_{0}}$ increases undeviating.

$\:\varvec{M}\varvec{o}\varvec{d}\varvec{e}\:\varvec{I}\varvec{I}\:[{\varvec{t}}_{1}-{\varvec{t}}_{2}]$: During this mode, switch$\:\:{S}_{1}$ remains in ON state, whereas switch $\:{S}_{2}$ turns OFF as depicted in Fig. 5(b). At this case, diode$\:{\:D}_{1}$ turns OFF and another diode$\:{\:D}_{2}$ turns ON. Subsequently, the energy from leakage inductor$\:{\:L}_{2}$ is observed by capacitor $\:{C}_{2}$ using $\:{D}_{2}$ diode, thereby which current across diode$\:\:{i}_{{D}_{2}\:}$starts decreasing. Consequently, the output inductance$\:{\:L}_{0}$ charges the load continuously and results in reduced inductor current$\:\:{i}_{{L}_{0}}$.

$\:\varvec{M}\varvec{o}\varvec{d}\varvec{e}\:\varvec{I}\varvec{I}\varvec{I}\:[{\varvec{t}}_{2}-{\varvec{t}}_{3}]$: During this condition switch $\:{S}_{1}$ occupies OFF position and the switch $\:{S}_{2}$ turns ON as illustrated in Fig. 5(c). Accordingly, $\:{D}_{1}$ diode turns ON and $\:{D}_{2}$ turns OFF. At the instance, energy from leakage inductance$\:{\:L}_{1}$ is absorbed by $\:{C}_{1}$ capacitor by $\:{D}_{1}$ diode and the corresponding current $\:{i}_{{D}_{1}\:}$ decreases in linear. Therefore, voltage across switch $\:{S}_{1}$ gets clamped at$\:\:{VC}_{2}$. As a consequence, the output inductor$\:{\:L}_{0}$ stores energy continuously resulting in increased inductance current$\:{\:i}_{{L}_{0}}$.

1 $$\:{V}_{in}={V}_{{L}_{1}}$$

2 $$\:{V}_{in}={V}_{{L}_{2}}$$

3 $$\:{V}_{{L}_{1}}={V}_{{L}_{2}}$$

4 $$\:{V}_{{C}_{2}}+{V}_{{L}_{0}}-{V}_{{C}_{0}}-{V}_{{C}_{1}}=0$$

5 $$\:{V}_{{C}_{0}}={V}_{0}$$

6 $$\:{V}_{{L}_{1}}-{V}_{{L}_{2}}-{V}_{{C}_{2}}=0$$

7 $$\:{V}_{{L}_{2}}={V}_{{L}_{1}}-{V}_{{C}_{2}}$$

8 $$\:{V}_{{L}_{0}}-{V}_{{C}_{0}-}{V}_{{C}_{1}}=0$$

9 $$\:{V}_{in}-{V}_{{L}_{1}}+{V}_{{C}_{1}}=0$$

10 $$\:{V}_{in}+{V}_{{C}_{1}}={V}_{{L}_{1}}$$

11 $$\:{V}_{{C}_{0}}-{V}_{{C}_{2}}-{V}_{{L}_{0}}-{V}_{{C}_{1}}=0$$

12 $$\:{V}_{{C}_{1}}-{V}_{{L}_{2}}-{V}_{{C}_{2}}-{V}_{{L}_{0}}-{V}_{{C}_{0}}=0$$

On subtracting Eq. (11) from (12)

13 $$\:{V}_{{L}_{1}}-{V}_{{L}_{2}}-2{V}_{{C}_{0}}=0$$

**Fig. 6:**
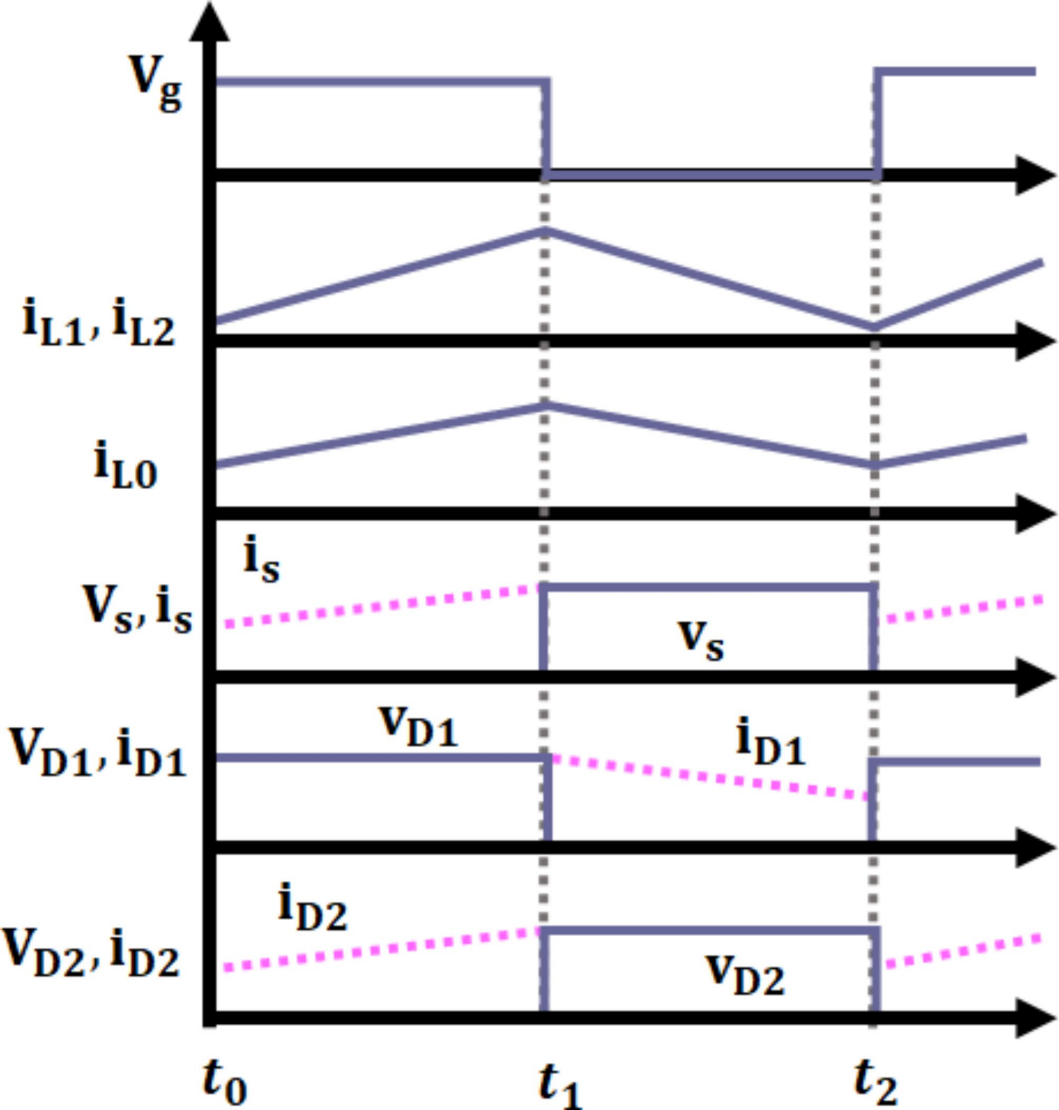
Switching waveform of proposed IBC converter.

$$\:\text{A}\text{p}\text{p}\text{l}\text{y}\text{i}\text{n}\text{g}\:{V}_{{C}_{0}}={V}_{0}\:\text{a}\text{n}\text{d}\:{V}_{{L}_{2}}={V}_{in}$$

14 $$\:{V}_{{L}_{1}}-{V}_{in}-2{V}_{0}=0$$

15 $$\:{V}_{{L}_{1}}={V}_{in}-2{V}_{0}$$

On applying voltage second balance Eq. 

16 $$\:\left({V}_{{L}_{1},\:{S}_{on}}\right)\left(DT\right)+\left({V}_{{L}_{1},\:{S}_{off}}\right)\left(1-D\right)T=0$$

By substituting Eqs. (1) and (15) in (16)

17 $$\:{V}_{in}\left(DT\right)+\left({V}_{in}+2{V}_{0}\right)\left(1-D\right)T=0$$

18 $$\:{V}_{in}DT+{V}_{in}T+2{V}_{0}T-{V}_{in}DT-2{V}_{0}DT=0$$

19 $$\:{V}_{in}T+2{V}_{0}T-2{V}_{0}DT=0$$

20 $$\:{V}_{in}+2{V}_{0}-2{V}_{0}D=0$$

21 $$\:{V}_{in}-2{V}_{0}(1+D)=0$$

22 $$\:{V}_{in}=2{V}_{0}(1+D)$$

Thereby, the gain of proposed IBC converter is expressed as

23 $$\:M=\frac{{V}_{0}}{{V}_{in}}=\frac{1}{2(1+D)}$$

Figure (6) shows the switching waveform of proposed Converter.

Henceforth, the proposed converter results with improved voltage gain. However, for optimal operation of converter control operation is essential. This is accomplished with the adoption of IMO cascaded ANFIS MPPT for attaining optimal Fuel cell voltage.

### Voltage stress

The voltage stress across capacitor $\:{C}_{1}$ and $\:{C}_{2}$ is expressed as ;

24 $$\:{V}_{vps}-{C}_{1}=\frac{1}{1-D}{V}_{in}=\frac{1}{3+D+2n}{V}_{0}=2.33V$$

25 $$\:{V}_{vps}-{C}_{2}=\frac{2}{1-D}{V}_{in}=\frac{2}{3+D+2n}{V}_{0}=3.20V$$

Where *n* = 1 and D = 0.6.

The voltage stress across Switches $\:{S}_{1}$ and $\:{S}_{2}$ is expressed as

26 $$\:{{V}_{vps}-{S}_{1}=V}_{vps}-{S}_{2}=\frac{1}{1-D}{V}_{in}=\frac{1}{3+D+2n}{V}_{0}=55V$$

Similarly, voltage stress across diode is given by,

27 $$\:{V}_{vps}-{D}_{1}=\frac{1}{1-D}{V}_{in}=\frac{1}{3+D+2n}{V}_{0}=0.453V$$

28 $$\:{V}_{vps}-{D}_{2}=\frac{2}{1-D}{V}_{in}=\frac{2}{3+D+2n}{V}_{0}=0.49V$$

### Loss analysis

The losses across switch involves switching loss and conduction loss$\:{\:C}_{loss}$, and the expression is given by;

29 $$\:{P}_{S}={I}_{rms-{S}_{1}}^{2}{r}_{{S}_{1}}+\frac{{f}_{s}}{2}\left[{V}_{D{S}_{1}}{I}_{{L}_{1}}{t}_{off1}+{V}_{D{S}_{1}}^{2}{C}_{loss1}\right]+{I}_{rms-{S}_{2}}^{2}{r}_{{S}_{2}}+\frac{{f}_{s}}{2}\left[{V}_{D{S}_{2}}({I}_{{L}_{1}}+{{I}_{0})t}_{off2}+{V}_{D{S}_{2}}^{2}{C}_{loss2}\right]=1220mW$$

Capacitor loss is expressed as;

30 $$\:{P}_{C}=\left(\sum\:_{k=\text{1,2}}{I}_{rms-{C}_{k}}^{2}{r}_{{C}_{k}}\right)+{I}_{rms-{C}_{0}}^{2}{r}_{{C}_{0}}=69.5mW$$

Hence, effective boosting of voltage is achieved and the duty cycles are adjusted by adopting IMO cascaded ANFIS for attaining optimal Fuel cell voltage.

### Improved may fly optimized cascaded anfis mppt controller

An improved control algorithm developed for MPPT in the proposed fuel cell devices is IMO Cascaded ANFIS MPPT Controller. It enhances the effectiveness and accuracy of MPPT in a variety of environmental circumstances by combining the Mayfly optimisation algorithm with a cascaded ANFIS. Here is an explanation of the controller’s main parts and how they work:

## Initialization of cascaded ANFIS MPPT controller

**Fig. 7:**
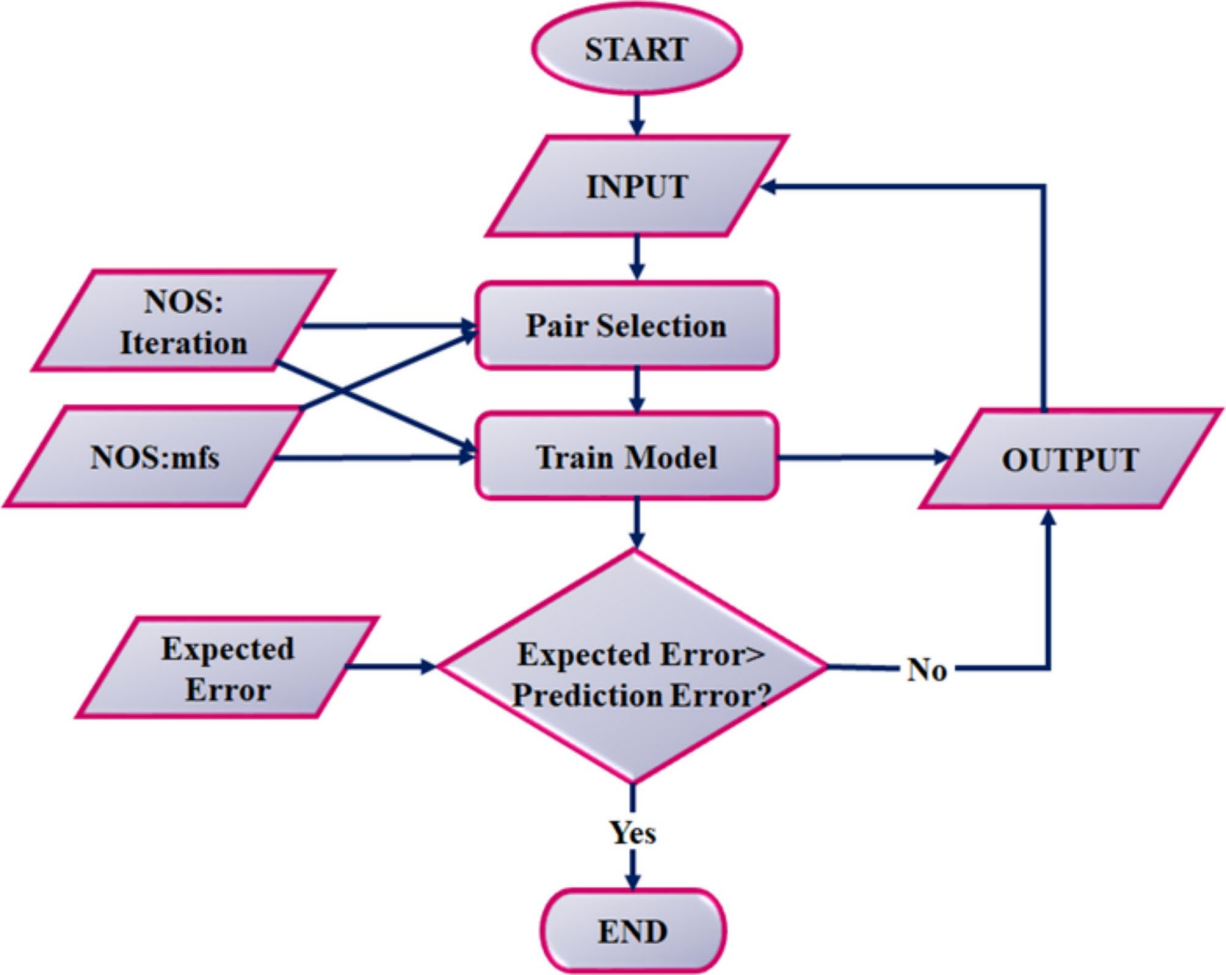
Structure of cascaded ANFIS.

The proposed work make use of cascaded ANFIS MPPT controller to maximize power output from PEMFC, by continuously adjusting the operating parameters. A flow description of cascaded ANFIS is found in Fig. 7. The main difference between novel algorithm and traditional ANFIS algorithm lies in the fact that output of traditional ANFIS algorithm becomes input for next usage. The initialization process and subsequent operation servers as a crucial steps, as follows:

### Initialization of primary ANFIS Controller

The ANFIS controller generated reference voltage and current depending on the current operational condition of FC. Key variables such as temperature, pressure and fuel cell output voltage and current are used as inputs. The input variables are fuzzified by means of membership function. These functions is responsible for transforming crisp input to fuzzy sets enabling the ANFIS to handle inherent uncertainty and non-linearity in fuel cell’s behaviour. At last set of fuzzy rules is created depending on historical data. These rules defines the relation between input variables related to output.

### Initialization of secondary ANFIS controller

The secondary ANFIS controller adjust the duty cycle of proposed IBC converter for ensuring fuel cell operates at its MPP. The reference values generated by the primary ANFIS controller, along with real-time measurements of voltage and current, serves as input to secondary ANFIS. Similar to primary ANFIS, the secondary controller uses fuzzification and a rule base to manage the converter’s duty cycle adjustments.

### Pair selection module

The objective here is to improve the accuracy of ANFIS model by selecting the best pairs of input variables. A sequential feature selection process is used, where each pair of input variables is evaluated based on their ability to minimize Root Mean Square Error (RMSE) between the predicted and actual outputs. For each pair, a two-input ANFIS model is trained and tested. The pair that results in the lowest RMSE is selected.

### Training module

The RMSE is calculated for each data pair, comparing the predicted outputs with actual outputs. The cascaded ANFIS model undergoes iterative training until RMSE falls below a predefined target error. The outputs from each iteration serve as inputs for the subsequent iteration, refining the model’s accuracy.

The Cascaded ANFIS model’s illustration approach is shown in Fig. 7. Assume that the optimisation issue designated has four input variables$\:\:{Z}_{1},{Z}_{2},{Z}_{3}\:and\:{Z}_{4\:}$, as shown here.

31 $$\:input=\{{Z}_{1},{Z}_{2},{Z}_{3},{Z}_{4\:}\}$$

The input is matched with best match defined in Eq. (31) below, as is described in pair selection Sect. 

32 $$\:{input}_{pairs}=\left\{{Z}_{1},{Z}_{3}\right\},\left\{{Z}_{2},{Z}_{1}\right\},{\{Z}_{3},{Z}_{4\:}\},\{{Z}_{4},{Z}_{1\:}\}$$

Henceforth, by two input models for every match two outputs are resulted as $\:{RMSE}_{i}$ and output predicted$\:\:{(Y}_{i})$.

33 $$\:RMSE=\sqrt{\stackrel{-}{{(A-P)}^{2}}}$$

34 $$\:{RMSE}_{A,P}={\left[{\sum\:}_{i=1}^{N}\frac{{({O}_{Ai}-{O}_{pi})}^{2}}{N}\right]}^{1/2}$$

35 $$\:f=\frac{{w}_{1}}{{w}_{1}+{w}_{2}}{f}_{1}+\frac{{w}_{2}}{{w}_{1}+{w}_{2}}{f}_{2}+\frac{{w}_{3}}{{w}_{2}+{w}_{3}}{f}_{3}+\frac{{w}_{4}}{{w}_{3}+{w}_{4}}{f}_{4}$$

From the expressions mentioned above predicted and actual outcomes is designated as$\:\:P\:and\:A$. Size and sample as$\:\:N$. Initial iteration is terminated by attained outputs from$\:\:Y\:and\:RMSE$. Now the RMSE and goal error have been compared, and the next iteration is chosen accordingly. The unique feature of this method is that when proceeding to the next iteration, the results from previous one $\:{Y}_{1},{Y}_{2},{Y}_{3}\:and\:{Y}_{4\:}$serves as inputs for subsequent iteration.

The same process is followed for extracting maximum power from fuel cell. During operation, the fuel cell variables are continuously monitored by the trained ANFIS modules. These modules predict the value for desired voltage and current, which is used to maximize the power output from fuel cell based on inputs. To maintain the system to operate at peak power, the controller adjusts the operating conditions accordingly. To optimize the parameters of cascaded ANFIS, the IMO algorithm is introduced.

### IMO algorithm for tuning cascaded ANFIS MPPT parameters

The IMO algorithm mimics the mating behaviour of mayflies employed to optimize the control settings of cascaded ANFIS for MPPT in fuel cell system. This approach combines, random, local and global search techniques to determine the optimal control parameters, maximizing power output from fuel cell.

**Fig. 8:**
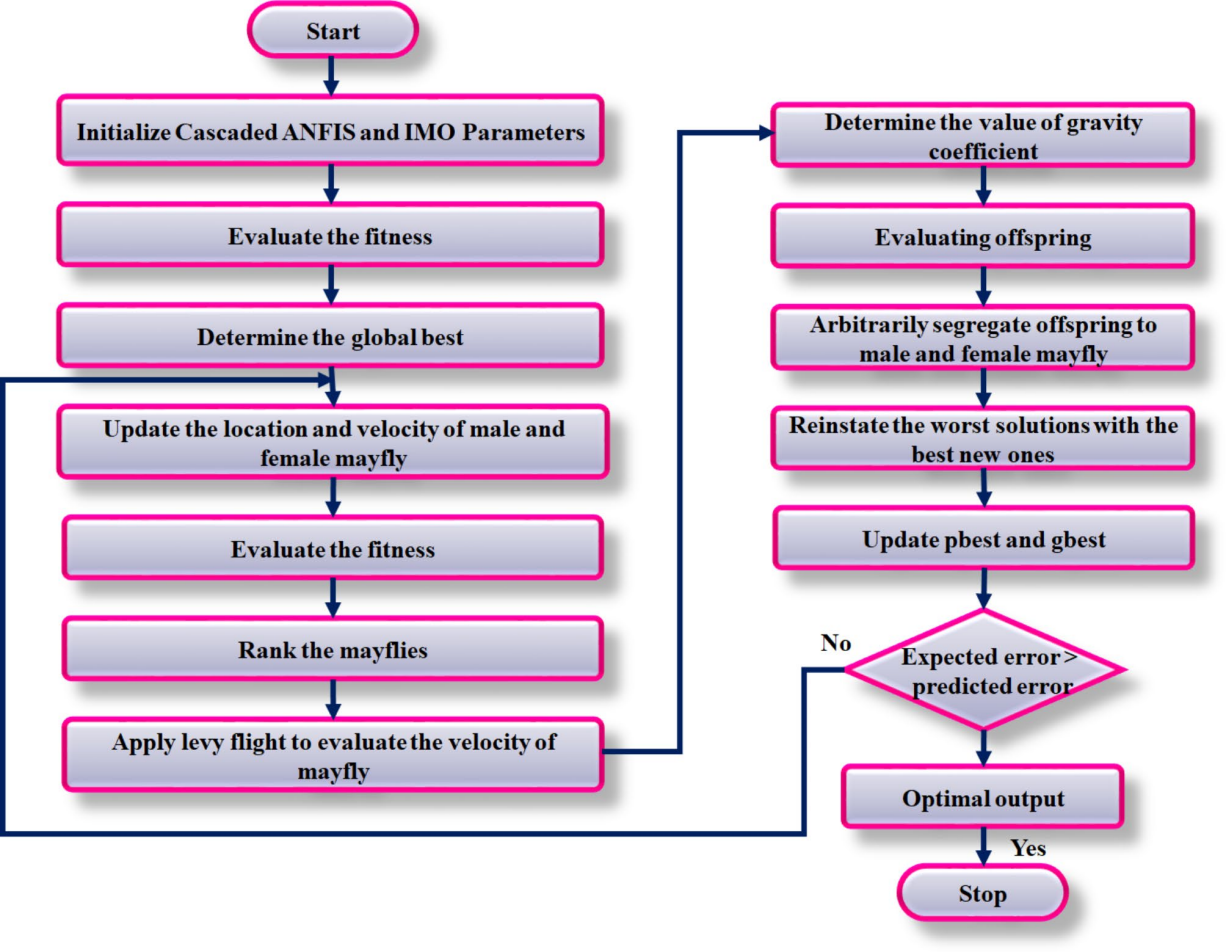
Flow chart of IMO cascaded ANFIS for MPPT.

**Table 2:** Parameter specification of IMO.

Parameter	Rating
$\:Size\:of\:Population$	$\:50$
$\:Maximum\:Iteration$	$\:500$
$\:Mutation\:Rate$	$\:0.1$
$\:Crossover\:Rate$	$\:0.8$

The cascaded ANFIS controller contains multiple layers of fuzzy inference system, each of which is responsible for monitoring and predicting voltage and current affecting the fuel cells output. At this instance, the population of mayflies are initialized with random positions and zero initial speed. The flow description of proposed IMO algorithm is represented in Fig. 8. Here, each of mayflies represents a potential solution for control of cascaded ANFIS parameter. The specification parameters of IMO algorithm is listed in Table 2. To ensure stability and effectual control, the parameters including membership function and weight are optimized within specific bounds. The IMO approach, in this stage defines the maximum speed $\:{v}_{max}$ for every mayfly, in order to prevent excess movement. Thereby, the algorithm focus toward optimal solution:

36 $$\:{v}_{max}=rand*({x}_{max}-{x}_{min})$$

Continuous adjustment of control parameter supports in keeping the fuel cell operate at MPP. Therefore, update the speed and position of each mayfly using local and global search approach, assisted by the best solution:

37 $$\:{v}_{i}^{k+1}=g*{v}_{i}^{k}+{a}_{1}{e}^{-\beta\:{r}_{p}^{2}}\left(p{best}_{i}-{x}_{i}^{k}\right)+{a}_{2}{e}^{-\beta\:{r}_{p}^{2}}(gbest-{x}_{i}^{k})$$

From Eq. (29) $\:g$ defines gravity coefficient, personal best position $\:pbest\:$as and global best position as$\:\:gbest$. Individual speed of female is represented by

38 $$\:{v}_{i}^{k+1}=\left\{\begin{array}{c}g*{v}_{i}^{k}+{a}_{2}{e}^{-\beta\:{r}_{mf}^{2}}\left({x}_{i}^{k}-{x}_{i}^{k}\right),\:f\left({y}_{i}\right)>f\left({x}_{i}\right)\\\:g*{v}_{i}^{k}+fl*r,\:f\left({y}_{i}\right)\le\:f\left({x}_{i}\right)\end{array}\right.$$

Ensuring that the system does not gets traps at local optima is essential for dynamic adjustment of parameters to explore new solution. In this stage, the mutation step is introduced to occasionally disrupt the current position of mayflies, encouraging exploration of search space.

39 $$\:{offspring}_{n}={offspring}_{n}+\sigma\:{N}_{n}\left(\text{0,1}\right)$$

From the aforesaid expression distribution function’s standard deviation is represented by$\:\:\sigma\:$, and mean with standard normal distribution as$\:\:{N}_{n}\left(\text{0,1}\right)$. This process supports the algorithm to escape local optima and identifies better solutions. Fine tuning of control parameters is needed for stabilization of system at MPP. Therefore, gradually minimize the random movement coefficient to focus search around best solutions found. This ensures convergence:

40 $$\:{d}_{k}={d}_{0}{\delta\:}^{k},0<\delta\:<1$$

41 $$\:{fl}_{k}={fl}_{0}{\delta\:}^{k},0<\delta\:<1$$

Thereby, the proposed IMO cascaded ANFIS MPPT tracks the optimal power from fuel cell. As a consequence, the converter performs optimally for powering the BLDC motor.

### BLDC motor

**Fig. 9:**
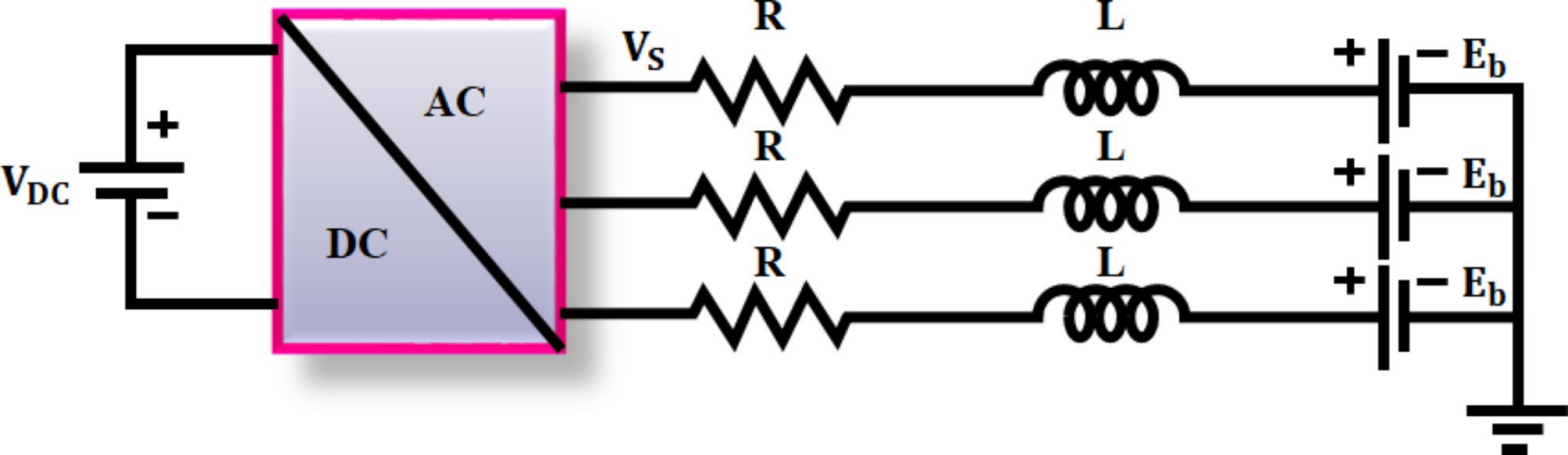
Equivalent configuration of BLDC motor.

The Brushless DC (BLDC) motor is a type of electrical motor that transforms electrical into mechanical energy. A $\:3{\Phi\:}\:$converter is necessary because this motor runs on $\:3{\Phi\:}$ AC source. It is possible to write the Eqs. (41) and (42) using circuit configuration depicted in Fig. 9.

The voltage equation per phase attained from BLDC motor is expressed as

42 $$\:{V}_{s}=R{I}_{a}+L\frac{{di}_{a}}{st}+{E}_{b}$$

in which, per phase stator resistance and inductance is represented as$\:\:R\:and\:L$, armature current as $\:{I}_{a}$ and back emf is specified as$\:\:{E}_{b}$. The electromagnetic torque accomplished by machine is determined as,

43 $$\:{T}_{e}=\frac{{3E}_{b}{I}_{a}}{\omega\:}$$

Hence, effective performance of EV motor is achieved by adopting IBC converter together with IMO cascaded ANFIS MPPT topology.

## Results and discussion

The examination of proposed topology is executed in MATLAB/Simulink environment with the designed parameters outlined in Table 3. Key performance metrics such as tracking accuracy, and response time are analyzed to demonstrate the superiority of proposed work over existing methods. Additionally, the impact of varying environmental conditions on the performance of system is examined to validate its robustness and reliability.

**Table 3:** Specification parameters.

Specification	Parameters
Fuel Cell Specification	
Maximum Power	$\:1\:KW$
Voltage	$\:90\:V$
Current	$\:11.11\:A$
Fuel Supply Pressure	$\:1.5\:\:bar$
Temperature	$\:338k$
**BLDC Motor Specification**	
Speed	$\:3000rpm$
Load Inertia	$\:9\times\:{10}^{-4}{Nm}^{2}$

**Fig. 10:**
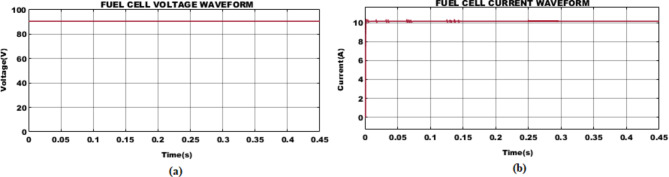
PEM fuel cell (**a**) voltage and (**b**) current waveform.

The voltage and current waveforms obtained from PEM fuel cell are illustrated in Fig. 10. To be noticed that, a stabilized fuel cell voltage of $\:90V$ is utilized for effective functioning of BLDC motor. Whereas current measuring $\:11.11A$ is attained after $\:0.25s$ with minor fluctuations at its initial stage.

**Fig. 11:**
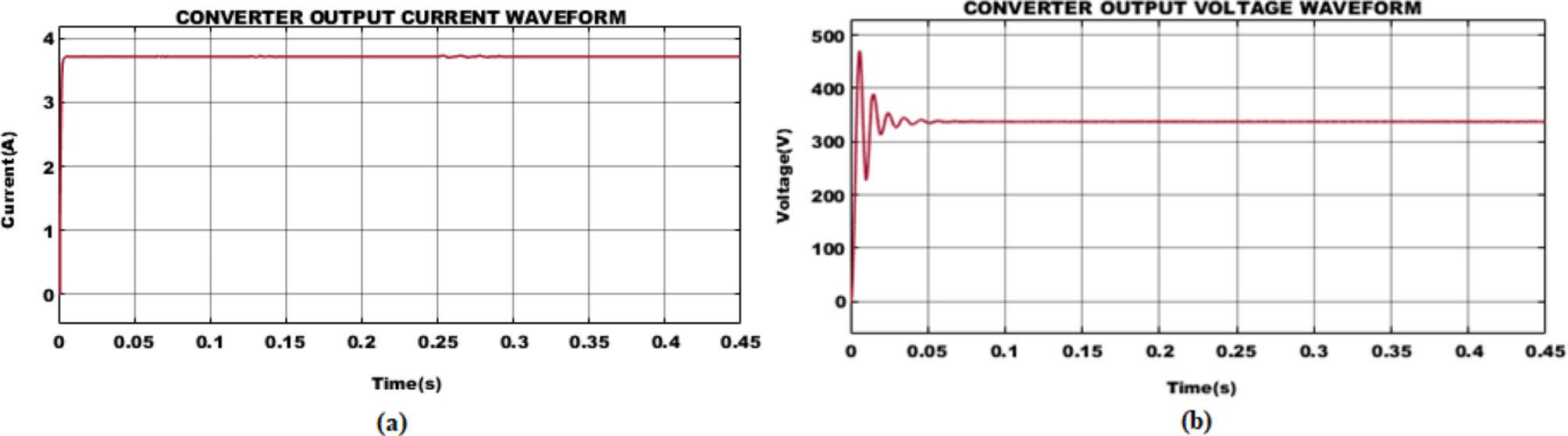
Interleaved boost-cuk converter (**a**) current and (**b**) voltage waveform.

The voltage from PV is effectively boosted for effective motor operation, utilizing IBC converter. The waveforms illustrating converter current, and voltage is depicted in Fig. 11. A stabilized current of $\:3.7A$ is sustained with minor distortions. Similarly, with voltage variation at the initial stage, improved voltage of $\:350V$ is attained after$\:\:0.1s$.

**Fig. 12:**
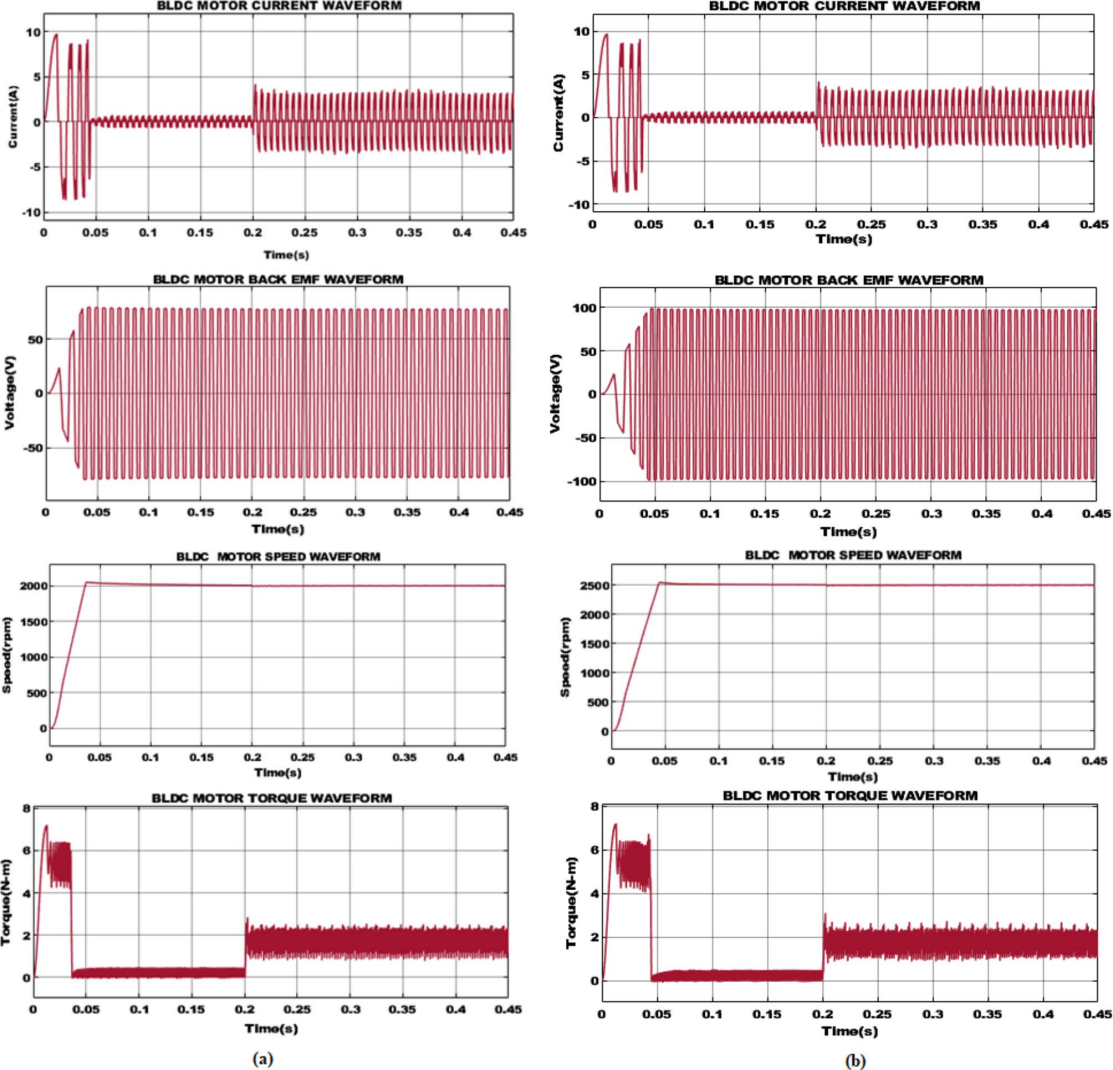
For speed (**a**) 2000 rpm and (**b**) 2500 rpm with Load 0.2s 1.5 Nm.

 The key waveforms observed from BLDC motor such as motor current, back EMF, motor speed and torque for varying speed is depicted in Fig. 12.

### Case 1: BLDC motor with 2000 rpm speed

The motor current initially, demonstrates oscillations and then stabilizes around 0.15s. The oscillation at initial phase is due to the dynamic response of motor to the applied load. After 0.2s, the motor current reaches a steady state and continues further with minimal fluctuations. Subsequently, the back EMF waveform demonstrates periodic oscillations, with respect to motor operation. The peak value of back EMF at 2000 rpm is consistent, indicating the stable operation of motor. The speed of motor ramps up to 2000 rpm, accomplishing desired speed shortly before 0.1s, indicating the rapid response of system. Finally, the torque waveform shows transients owing to sudden load application. The torque stabilizes around 0.2s, when 1.5Nm load is applied. This combination demonstrates the effectiveness of proposed system in handling load, as shown in Fig. 12(a).

### Case 2: BLDC motor with 2500 rpm speed

The waveform in Fig. 12(b) demonstrates the performance of BLDC motor operating at speed of 2500 rpm with same load of 1.5Nm applied at 0.2s. Accordingly, the motor current shows initial oscillations and then stabilizes around 0.15s. However, at 2500 rpm, the current magnitude is slightly higher compared to 2000 rpm. The back EMF waveform at 2500 rpm demonstrates periodic oscillations with peak value higher than 2000 rpm. The speed of motor rises rapidly to 2500 rpm, reaching set speed before 0.1s. Moreover, the torque waveform shows initial transients owing to load at 0.2s. This indicates that the motor effectively handles the load at 2500 rpm.

### Hardware analysis

**Fig. 13:**
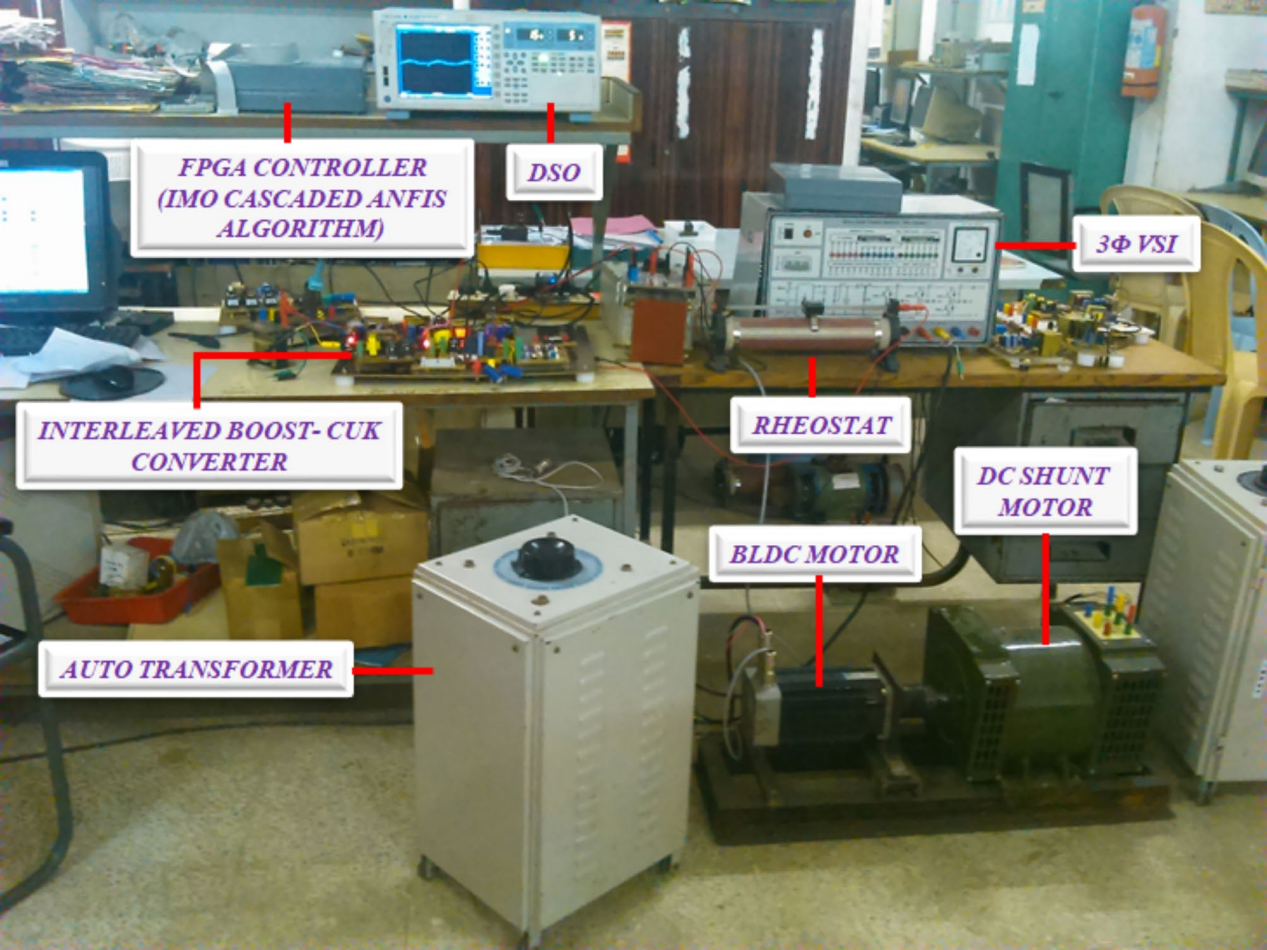
Experimental setup.

The hardware prototype seen in Fig. 13, is established by connecting a power converter that supplies a resistive load to a fuel cell simulator. Rapid prototype with SPARTAN 6E FPGA controller is utilized to implement MPPT algorithm and converter control. The SPARTAN 6E FPGA controller is programmed to generate precise and tailored logic pulses. These pulses serve as control signals for PWM generator. By manipulating the width of these pulses, FPGA effectively control the output power of converter in the system.

**Fig. 14:**
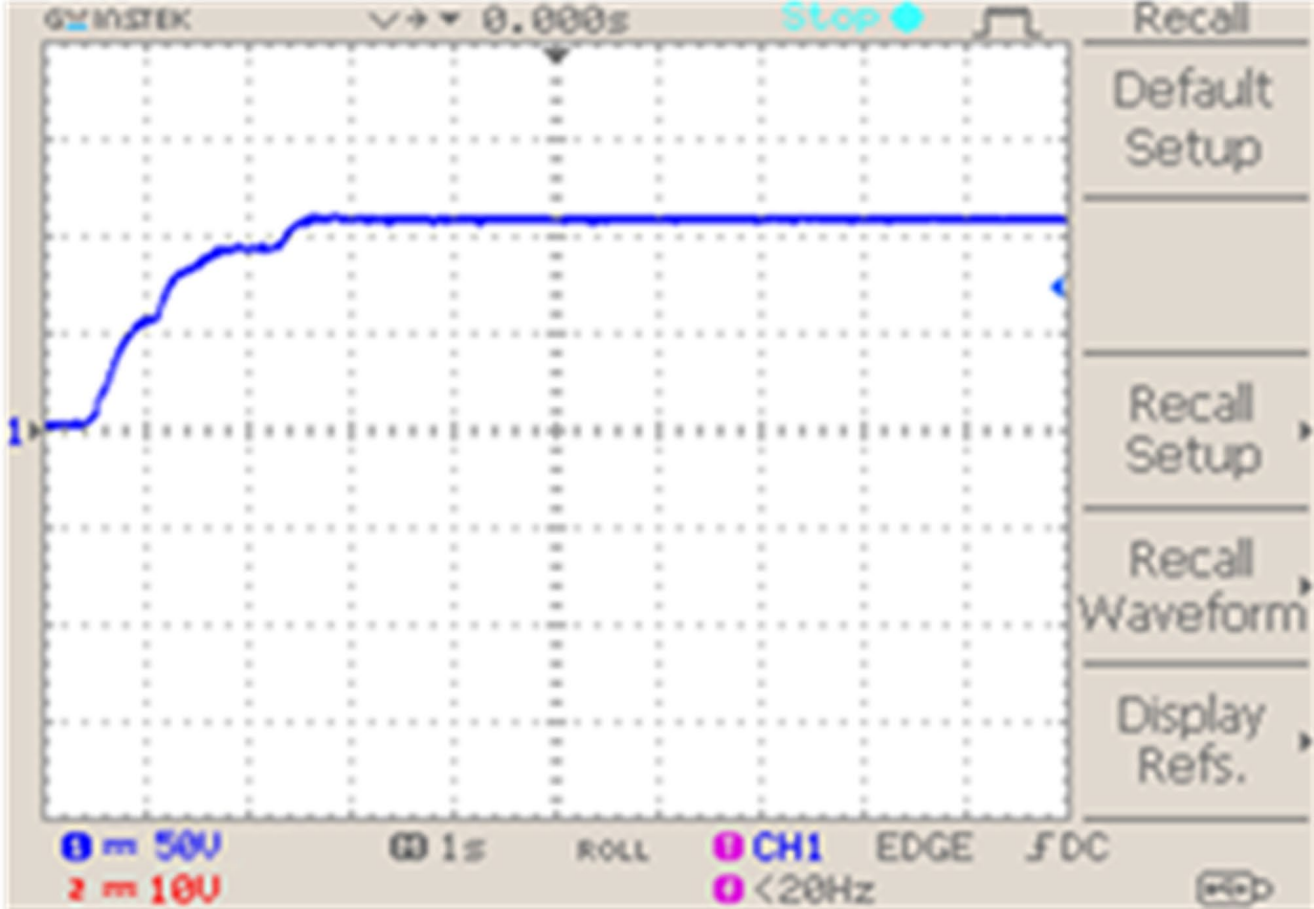
Fuel cell voltage.

The voltage across fuel cell in Fig. 14 demonstrating that with initial variation a stabilized voltage 110 V is maintained, consistently throughout operation. This is essential for effective functioning of fuel cell.

**Fig. 15:**
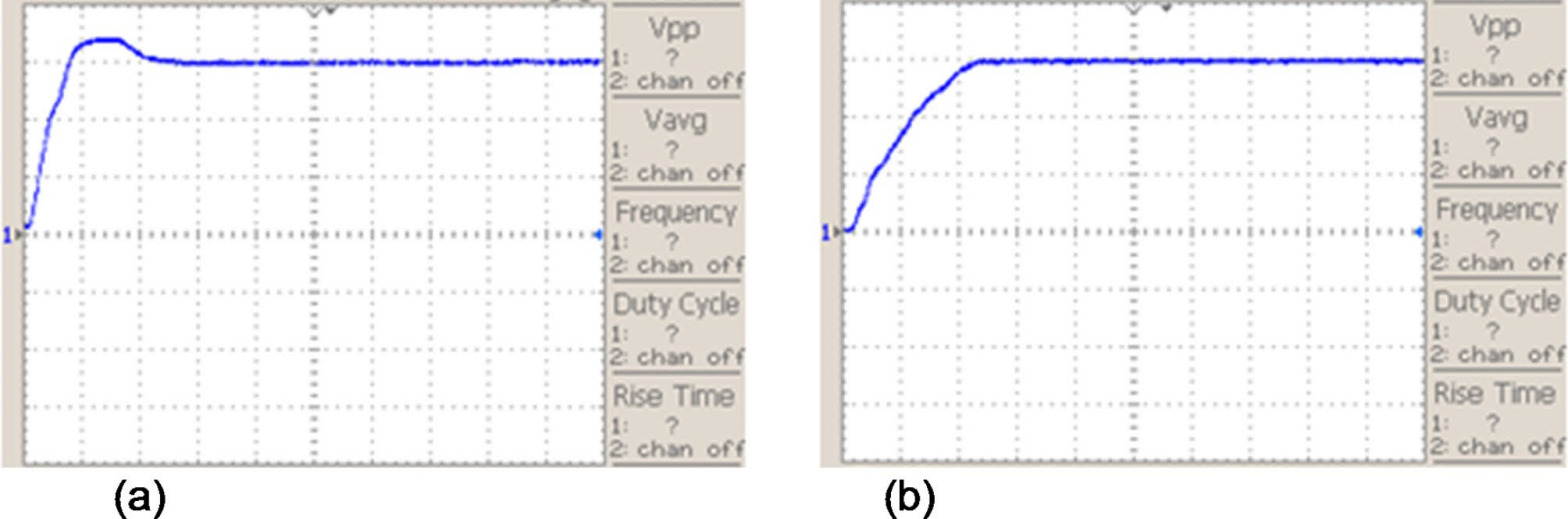
Interleaved boost cuk converter output voltage using (**a**) ANFIS controller and (**b**) IMO cascaded ANFIS controller.

In Fig. 15 graph shows how an interleaved Boost-Cuk converter operated by two distinct control techniques responds to changes in output voltage:

- ANFIS Controller: Uses an Adaptive Neuro-Fuzzy Inference System (ANFIS) controller to represent the output voltage response.
- IMO Cascaded ANFIS Controller: Displays the result of using a cascaded ANFIS controller that has been adjusted using Improved Metaheuristic Optimisation (IMO).

The analysis of different MPPT approach is represented in Fig. 14, in extracting the most power from fuel cell. The results in Fig. 15, indicate that compared to ANFIS controller the proposed IMO cascaded ANFIS results with improved converter performance. This improvement supports in effective operation of fuel cell system connected to BLDC motor.

**Fig. 16:**
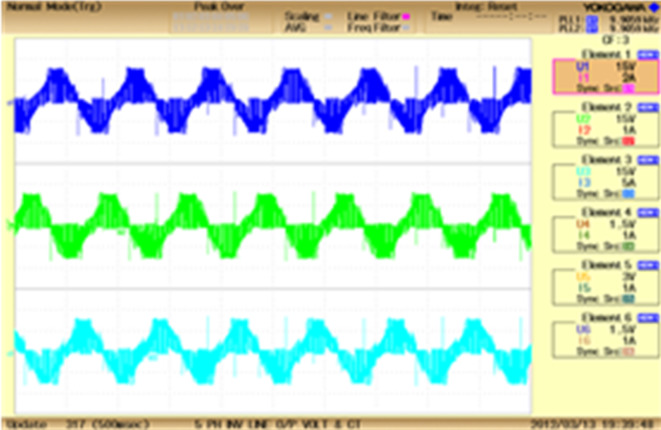
$$\:3\varphi\:$$ VSI output.

The output voltage waveform of $\:3\varphi\:$ VSI is explained in Fig. 16, which also shows the presence of back EMF. The converter has been operated for 320 V with the switching frequency of 10 kHz.The VSI performed the operation with the acceptable THD of 9.6%.The source voltage becomes trapezoidal due to the presence of back EMF.

**Fig. 17:**
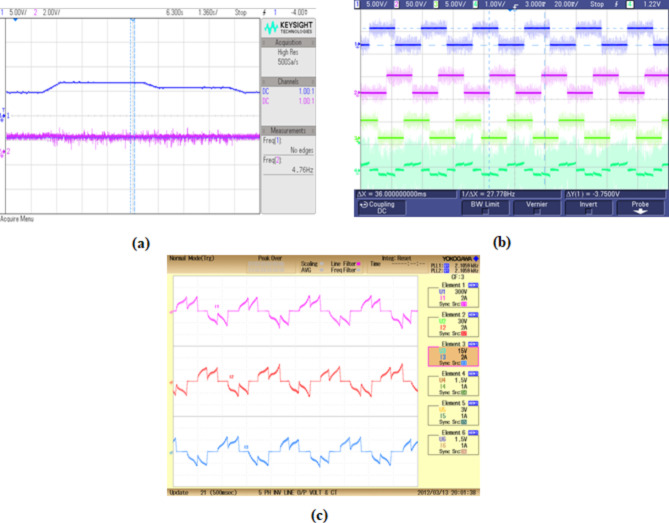
BLDC motor (**a**) torque waveform (**b**) hall sensor signal and stator current waveform and (**c**) output current waveform.

 The torque waveform of BLDC motor show in Fig. 17(a) demonstrates torque owing motor operation. The waveform indicates the motor response to load conditions. Followed by Fig. 17(b) presenting the hall sensor signals and stator current waveform demonstrating the synchronized operation of hall sensors and stator current ensuring efficient motor performance with minimal losses. Subsequently in Fig. 17(c), output current of motor is represented showing consistent current level with minor fluctuations.

**Fig. 18:**
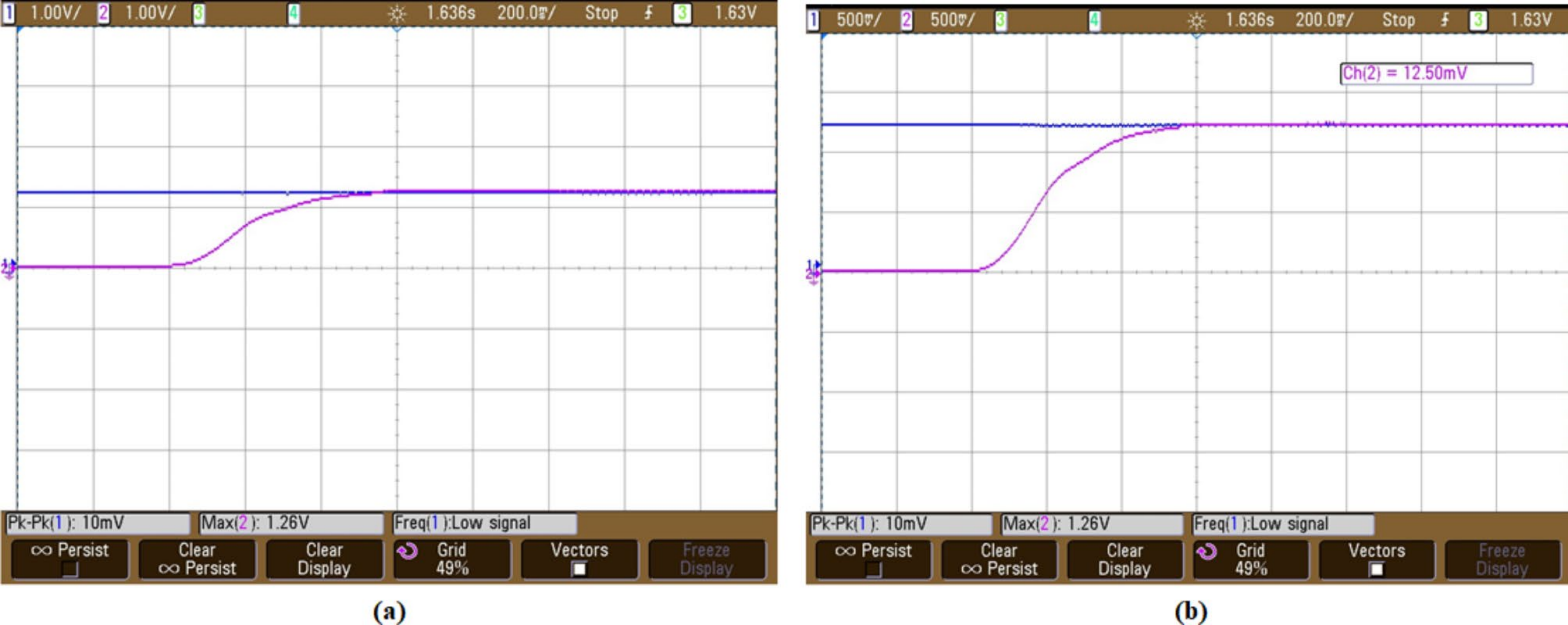
Speed waveform using PI controller (**a**) 1500 rpm and (**b**) 2500 rpm.

 The speed waveform of BLDC motor utilizing PI controller is shown in Fig. 18, with speed of 1500 rpm and 2500 rpm. The waveforms demonstrate that, initially, there is a rapid increase in speed, after it continues without any deviation. Similar to lower speed scenario, the waveform in Fig. 18(a),(b) shows the performance of motor accelerating towards the desired speed of 2500 rpm. This indicates the speed performance of motor using PI controller offering reliable and efficient control, under varying operating condition.

**Fig. 19:**
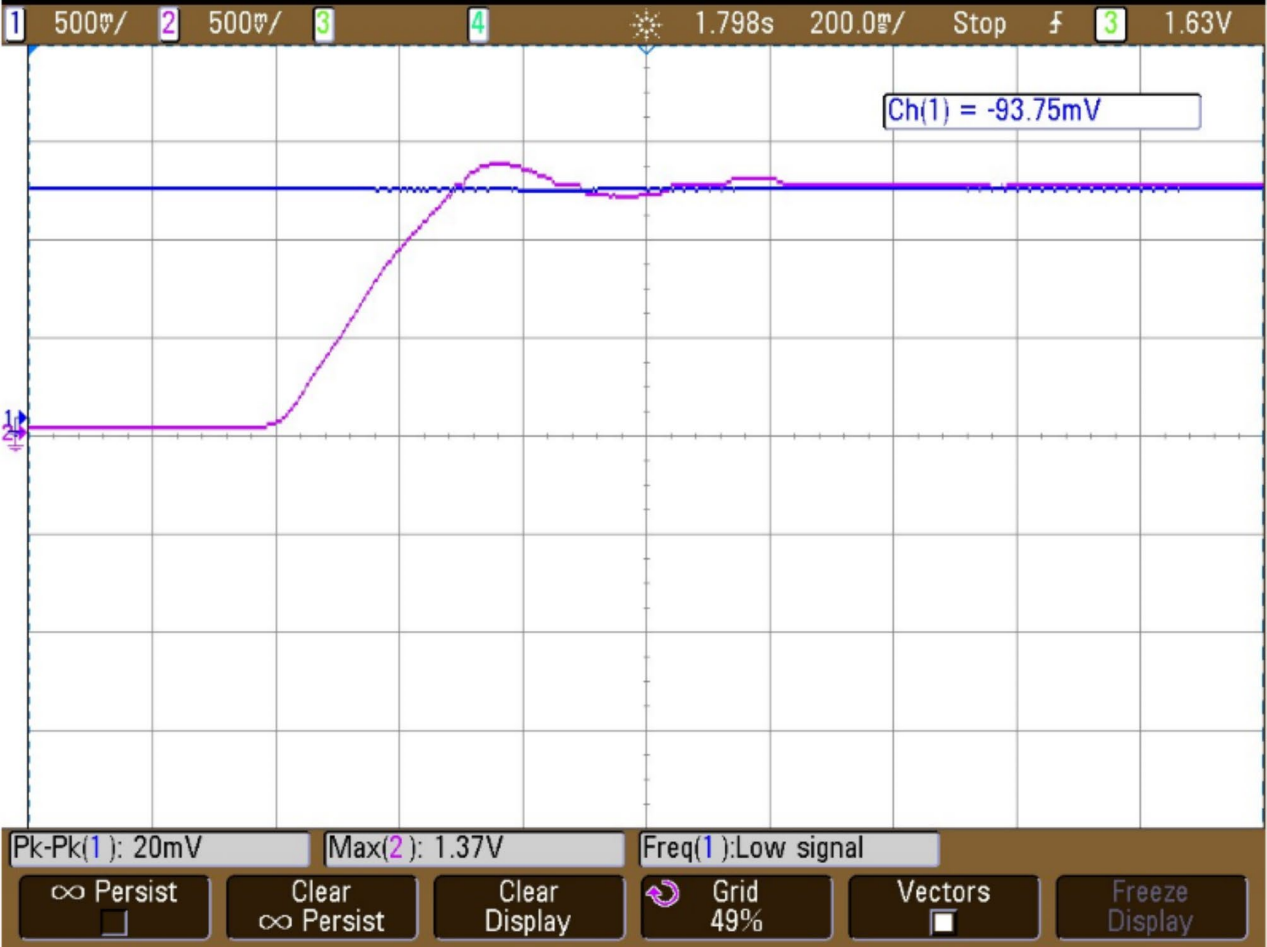
Speed waveform under non-uniform condition.

Figure 19 demonstrates the speed waveform of BLDC motor at non-uniform conditions, indicating the motor experience with severe oscillation, but this oscillation get stabilized over time due to the proposed control strategy. This strategy effectively minimizes the oscillations and supports the motor to stabilize at stable speed, detailing the improved performance and stability under varying condition. For a better understanding of proposed converter and BLDC motor simulation and hardware properties comparison has been presented in Table 4.

**Table 4:** Comparison of simulated and hardware results.

S.no	Parameter	Simulated		Hardware	
		Voltage (V)	Current (A)	Voltage (V)	Current (A)
1	Fuel cell	90	11.11	110	12
2	Proposed converter	350	3.7	348	3
**Performance of Converter with different controllers**					
3	ANFIS	420 V		385 V	
4	IMO	350 V		348 V	
**BLDC Information**					
5	Torque	1.5 N-m		1.5 N-m	
6	Efficiency	94%		91.42%	

**Fig. 20:**
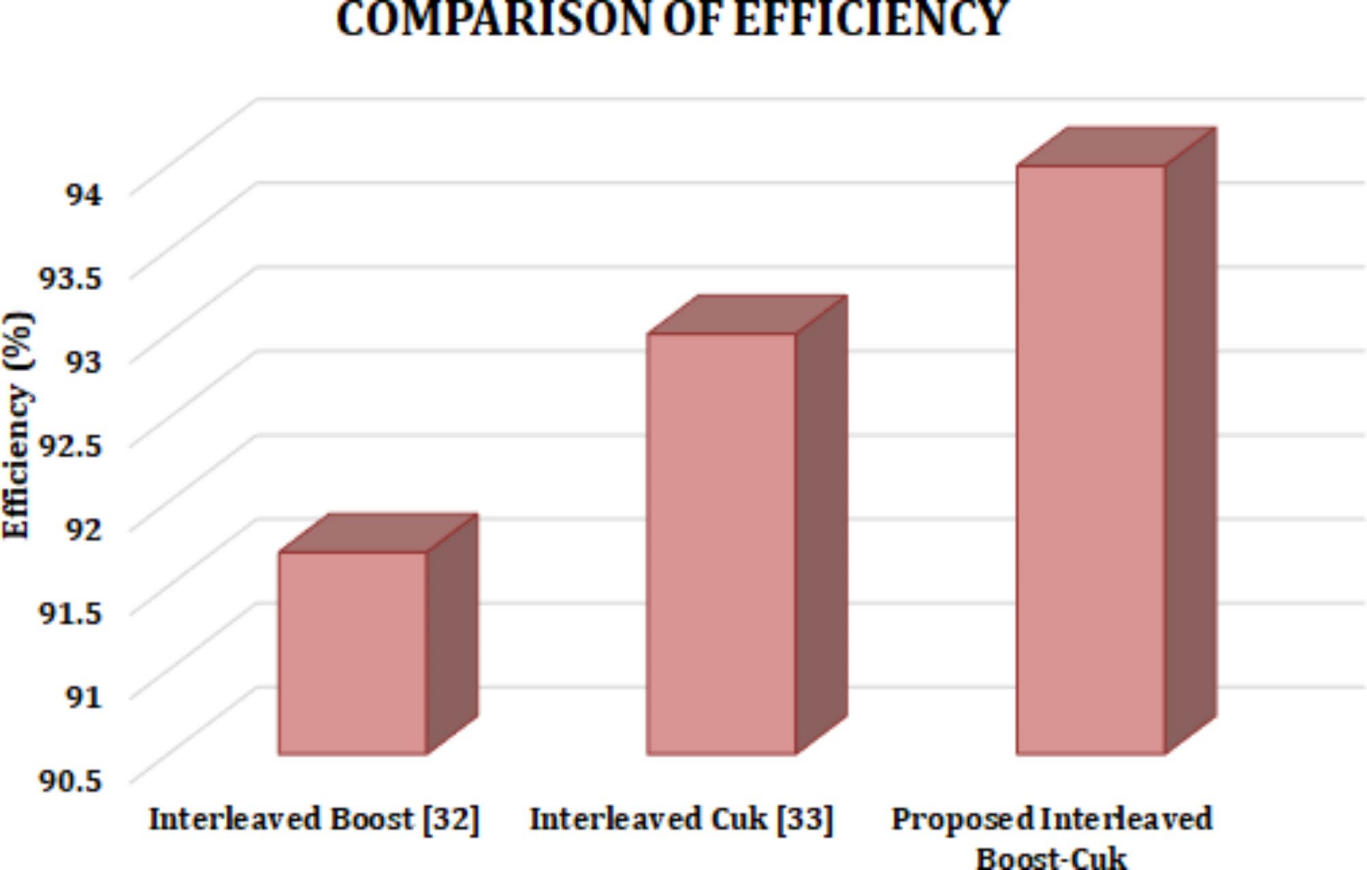
Comparison of converter voltage gain and efficiency.

The comparison of converters in terms of component count and efficiency is depicted in Table 5 and the corresponding graphical representation for efficiency of proposed converter is shown in Fig. 20. It is observed that proposed converter achieves improved efficiency of 94%, while the 2 ϕ IBC Nahar et al.^34^ 1st-M IBC Jang et al.^35^ Zhou et al.^36^ Varesi et al.^37^. interleaved Boost Li et al.^32^ and interleaved Cuk Joseph et al.^33^ converter shows reduced efficiency of 91.7% and 93%, respectively.

**Table 5:** Comparison of converter.

Converters	No. of Switches	No. of Capaci- tors	No. of Diodes	No. of Inductors	Voltage gain	Efficiency (%)
2 ϕ IBC^36^	2	1	2	2	1· 11	97·08
1st-M IBC^37^	2	2	2	2	1· 11	98·36
2nd-M IBC^38^	2	3	3	2	1· 11	98·99
3rd-M IBC^39^	3	3	3	2	1· 11	99·09
Interleaved Boost^32^	2	2	3	2	5·83	91.7
Interleaved Cuk^33^	2	4	2	4	5	93
Proposed	2	2	2	3	3·8	94

To determine the tracking efficiency of proposed controller various MPPT approaches are contrasted and listed in Table 6, of which the proposed IMO Cascaded ANFIS controller shows enhanced tracking efficiency of 98.92% when compared with approaches like Fuzzy^40^, APO^41^, ANFIS-PSO^42^.

**Table 6:** MPPT efficiency comparison.

MPPT Technique	Efficiency (%)
Fuzzy^40^	97.79
APO^41^	97.86
ANFIS-PSO^42^	98.35
IMO Cascaded ANFIS	98.92

### Comparative analysis

**Fig. 21:**
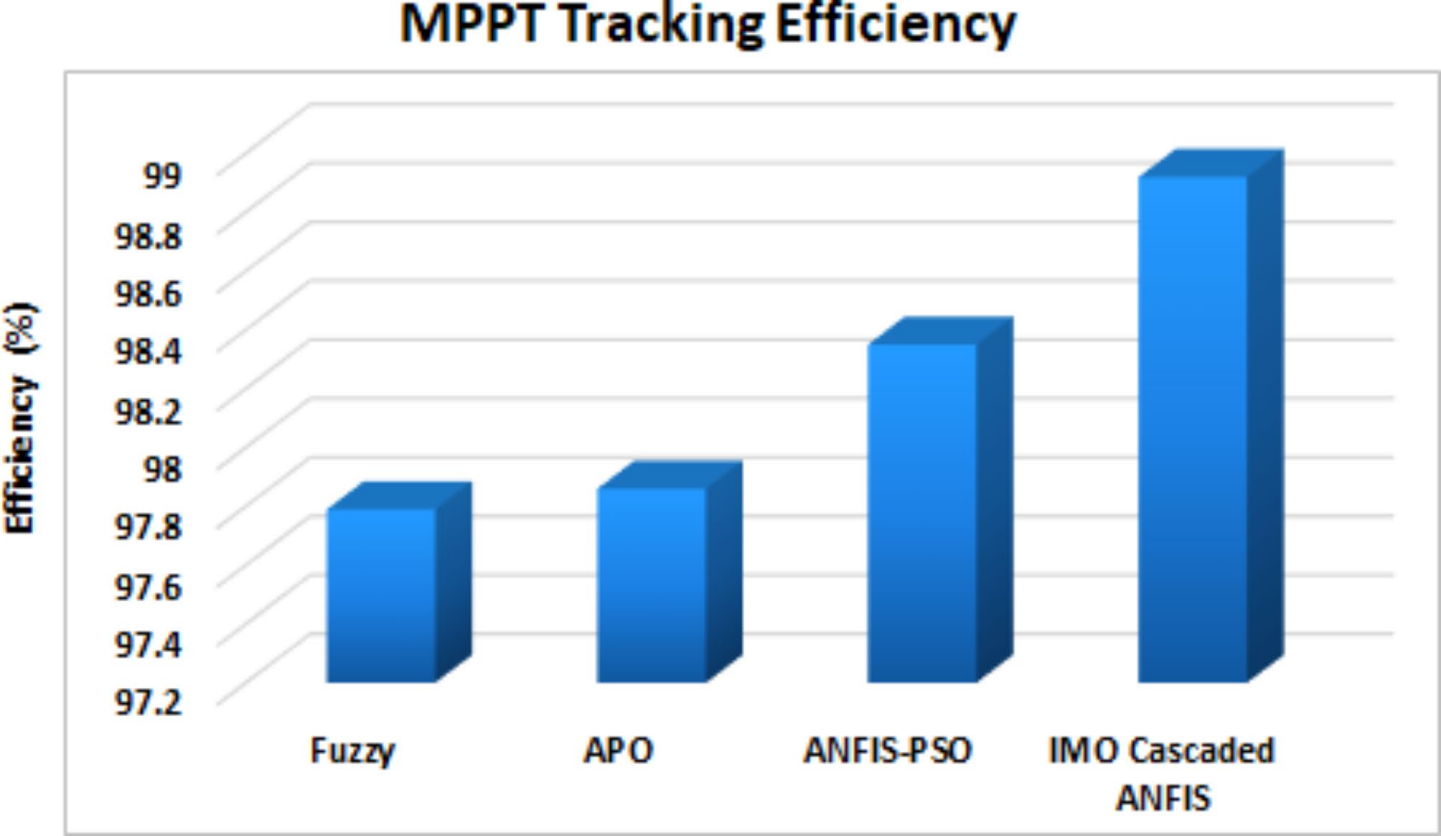
MPPT controller comparison.

 The tracking efficiency of different MPPT controller is depicted as graphical representation in Fig. 21. From Figure it is made clear that IMO Cascaded ANFIS controller results better in extracting the most possible power from PEM fuel cell when contrasted with other MPPT approaches.

**Fig. 22:**
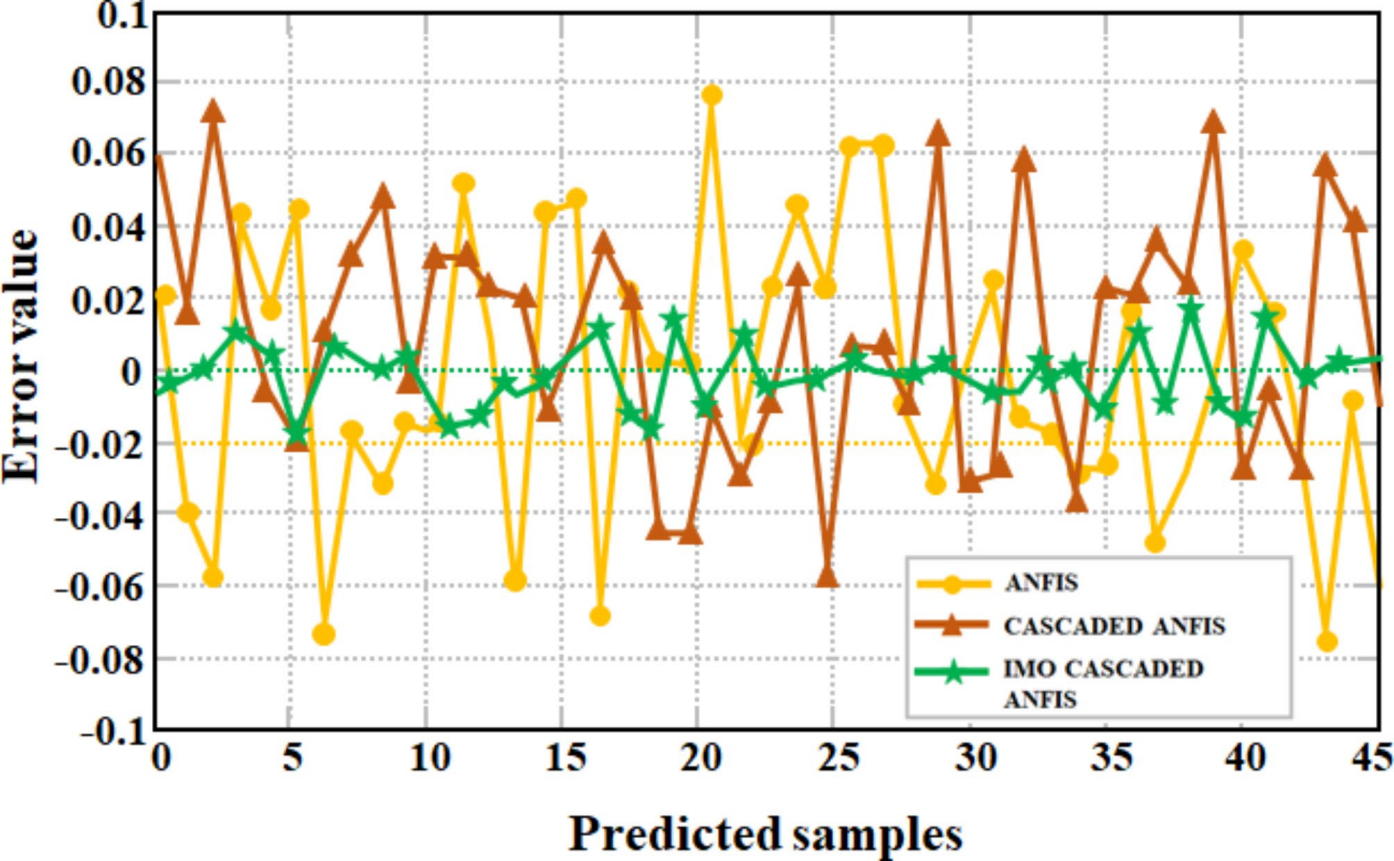
MPPT error comparison.

The comparison of prediction errors between ANFIS, Cascaded ANFIS and proposed IMO Cascaded ANFIS MPPT algorithms is illustrated in Fig. 22. This observation effectively highlights the differences between the predicted and actual observed values using diverse approaches. Notably, the IMO Cascaded ANFIS MPPT method stands out by showcasing significantly reduced error values, demonstrating its superior accuracy and predictive performance when compared to the conventional technique.

**Fig. 23:**
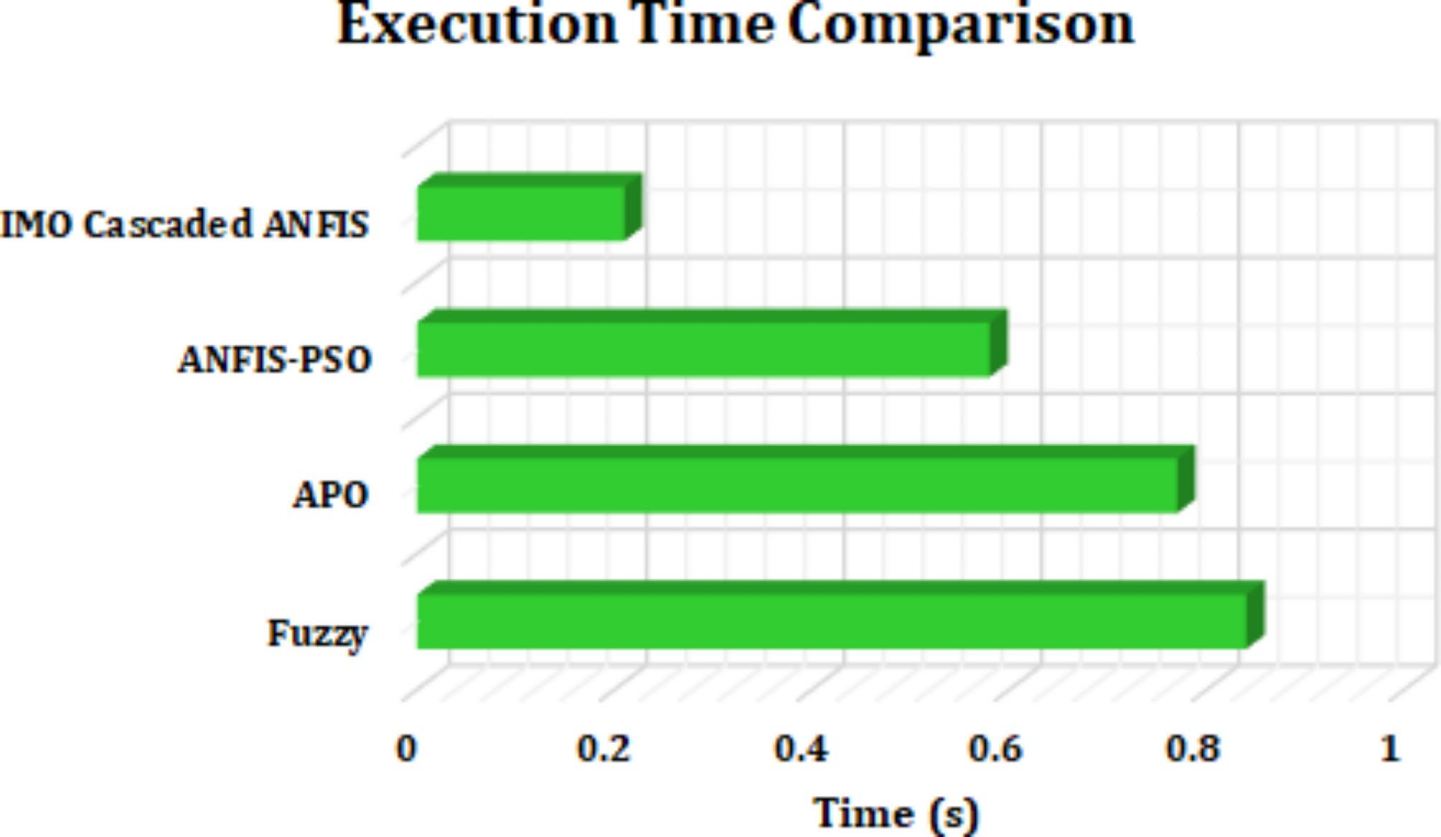
Comparison of execution time.

The execution time comparison for various MPPT approaches is illustrated in Fig. 23. It is noticed that Fuzzy approach^34^, has the highest execution time of 0.84s, with APO^35^, demonstrating moderate execution time of 0.77s. The ANFIS-PSO^36^ technique reveals improved execution time by 0.58s, allowing for faster convergence. Overcoming the mentioned techniques the proposed IMO cascaded ANFIS stands out with relatively lower execution time of 0.21s, owing to the advanced optimization strategy. This makes the proposed IMO cascaded ANFIS most efficient and suitable for tracking optimal power within shorter duration.

## Conclusion

This research presents a comprehensive approach for enhancement of efficiency and reliability of FC based EVs, with the integration of advanced power control technologies. With the proposed novel IBC converter, the PEMFC voltage gets boosted up to the requirement of BLDC motor. The proposed converter results with reduced switching losses and a high voltage gain conversion ratio. In addition, IMO cascaded ANFIS controller is proposed for optimal extraction of power from fuel cell, ensuring higher reliability and significance in improved charging efficiency. The potential of proposed work is validated through MATLAB simulations, demonstrating improved converter efficiency of 94%, outperforming other similar converter topologies. Additionally, the proposed IMO cascaded ANFIS MPPT exhibits superior tracking efficiency of 98.92% with execution time of 0.21s. Thereby, the proposed research contribute towards robust and efficient control mechanisms, leading for future advancements in motor operation.

## Data Availability

The data used to support the findings of this study are included in the article.

## Abbreviations

FC: Fuel cell
MPPT: Maximum power point tracking
PEMFC: Proton exchange membrane fuel cell
EV: Electric vehicle
IBC: Interleaved boost cuk converter
IMO: Improved Mayfly optimization
ANFIS: Adaptive neuro-fuzzy inference system
GHG: Greenhouse gas
ESS: Energy storage system
RES: Renewable Energy source
MWC: Membrane water content
P & O: Perturbation & Observation
INC: Incremental conductance
FLC: Fuzzy logic control
ANN: Artificial neural network
BLDC: Brushless direct current
PSO: Particle swarm optimization
RBFN: Radial basis function network
RMSE: Root mean squared error
APO: Augmented perturb & observe
